# Measuring human trace fear conditioning

**DOI:** 10.1111/psyp.14119

**Published:** 2022-06-08

**Authors:** Jelena M. Wehrli, Yanfang Xia, Samuel Gerster, Dominik R. Bach

**Affiliations:** ^1^ Computational Psychiatry Research, Department of Psychiatry, Psychotherapy, and Psychosomatics, Psychiatric Hospital University of Zurich Zurich Switzerland; ^2^ Wellcome Centre for Human Neuroimaging & Max Planck UCL Centre for Computational Psychiatry and Ageing Research University College London London UK

**Keywords:** calibration, fear conditioning, fear memory, fear‐potentiated startle, human neuroscience, psychophysiological modeling, retrodictive validity, trace fear memory

## Abstract

Trace fear conditioning is an important research paradigm to model aversive learning in biological or clinical scenarios, where predictors (conditioned stimuli, CS) and aversive outcomes (unconditioned stimuli, US) are separated in time. The optimal measurement of human trace fear conditioning, and in particular of memory retention after consolidation, is currently unclear. We conducted two identical experiments (*N*_1_ = 28, *N*_2_ = 28) with a 15‐s trace interval and a recall test 1 week after acquisition, while recording several psychophysiological observables. In a calibration approach, we explored which learning and memory measures distinguished CS+ and CS− in the first experiment and confirmed the most sensitive measures in the second experiment. We found that in the recall test without reinforcement, only fear‐potentiated startle but not skin conductance, pupil size, heart period, or respiration amplitude, differentiated CS+ and CS−. During acquisition without startle probes, skin conductance responses and pupil size responses but not heart period or respiration amplitude differentiated CS+ and CS−. As a side finding, there was no evidence for extinction of fear‐potentiated startle over 30 trials without reinforcement. These results may be useful to inform future substantive research using human trace fear conditioning protocols.

## Abstract 2

Our report generates new insights into the psychophysiology of trace fear conditioning using long trace intervals (15 s) in humans. By employing a strong explorative/confirmative strategy and comparing multiple psychophysiological measurements we identify skin conductance and pupil size responses to reflect fear acquisition and in agreement with previous research on delay fear conditioning show startle eye‐blink responses to reflect fear retention even a week later.

## 1 INTRODUCTION

Fear conditioning is a standard laboratory model for investigating the neurobiology of aversive learning (LeDoux, 2014), and for preclinical research into the modification of unwanted aversive memory (Milad & Quirk, 2012). In this paradigm, a conditioned stimulus (CS+) is contingently followed by an aversive unconditioned stimulus (US). Humans and many non‐human species learn to predict the US and express this prediction in preparatory behavior (Davis, 1992; Fanselow, 1994). A common instantiation of this approach in non‐human (Kochli et al., 2015) and human (Lonsdorf et al., 2017) experiments is delay fear conditioning, where the CS and US overlap in time. Such overlap, however, is not the case in many biological environments (e.g., lightning precedes thunder). Similarly, in the phenomenology of post‐traumatic stress disorder, remote cues that preceded the trauma can come to elicit arousal and memory intrusions (Ehlers et al., 2002). This situation is modeled by trace fear conditioning where CS and US are separated in time (Mertens et al., 2020; Rescorla, 1988; Sehlmeyer et al., 2009). While delay and trace fear conditioning are procedurally very similar, there are major differences in the neural circuits that support both types of learning, in particular an essential involvement of the hippocampus in trace fear conditioning as revealed in rodent work (Gilmartin et al., 2012).

Hence, it appears important to test candidate procedures for memory modification in trace fear conditioning protocols. However, the most appropriate quantification of the CS‐US association in human trace fear conditioning is currently unclear, partly because it is unknown which conditioned responses are elicited, and at what point in time they are expressed. While it has been observed that trace conditioning produces weaker responses than delay conditioning (Bouton, 2007), it is debated whether this reflects weaker learning (Raybuck & Lattal, 2014). Indeed, it has been suggested that the expression of trace conditioning in behavior might be different from the expression of delay conditioning, and might appear weaker because quantification strategies were optimized for delay conditioning (Raybuck & Lattal, 2014). For example, in delay conditioning where conditioned responses are well characterized (see Ojala & Bach, 2020 for a comprehensive review), varying the time interval between CS and US onset in delay conditioning can shift the expression of the CS‐US association in time (Castegnetti et al., 2016, 2017). This is likely to be of even more importance for trace fear conditioning.

Typical trace intervals in previous human trace fear conditioning studies were on the order of 1–10 s (Sehlmeyer et al., 2009). Neuroimaging has been used to verify hippocampal involvement in such paradigms. Hippocampal fMRI responses are reported both in delay and trace fear conditioning, with no confirmed dependency on the duration of the trace interval (Sehlmeyer et al., 2009). Also, it is not clear whether this hippocampal activation reflects CS‐US conditioning, or other processes such as concurrent episodic memory formation (Fullana et al., 2016). Using a wider range of trace intervals, non‐human animal studies have reported that hippocampal involvement in trace fear conditioning depends on the trace interval (Chowdhury et al., 2005; Misane et al., 2005). Chowdhury et al. (2005) demonstrated that lesions to the dorsal hippocampus only attenuated freezing after training at a 20 s trace interval, but not a a 1–3 s trace interval. Misane et al. (2005) found that by injecting the NMDA (N‐methyl‐D‐aspartate receptor) receptor antagonist APV ([2R]‐amino‐5‐phosphonovaleric acid) bilaterally into the dorsal hippocampus before fear conditioning led to impaired conditioned freezing in an assessment 24 h after training at trace intervals of 15 and 30 s, but not at shorter trace intervals of 1–10 s. Thus it seems that in rodents, the hippocampus is necessarily required for conditioning only with trace intervals longer than 10 s (Chowdhury et al., 2005; Misane et al., 2005), which have not typically been used in humans. Although neurobiological insights garnered from rodent experiments do not always extrapolate to humans, these results motivated a trace interval of 15 s for our experiments. To reduce potential backward conditioning, we used a longer inter‐trial interval of 30 s, during which participants performed an incidental task.

Our paradigm and the observables we recorded were based on delay fear conditioning research. We used visual CS and electric shock as US. Previous work has identified fear‐potentiated startle (Blumenthal et al., 2005) as a measure with high sensitivity to quantify fear memory retention after consolidation (Bach & Melinscak, 2020; Khemka et al., 2017), but the startles probes required for this measure can interfere with fear acquisition (de Haan et al., 2018; Sjouwerman et al., 2016). Hence, we recorded skin conductance, pupil size, electrocardiogram (ECG) and respiration during acquisition. During the recall session, 1 week after acquisition and without US, we elicited startle eye‐blink responses (SEBR), and recorded orbicularis oculi electromyogram (EMG), together with the same measures as during acquisition. As we aimed to apply the methods developed here for memory modification research, which usually employs a separate memory recall session after an intervention, our focus was the quantification of memory retention after overnight consolidation. With this goal in mind, our focus was to find the optimal measure for each session, rather than identify measures that are comparable across sessions.

We sought to characterize conditioned responses and develop an analysis scheme in a first exploratory experiment, and confirm these results in a second, independent sample. Our validation criterion was retrodictive validity, i.e., the sensitivity to distinguish CS+ and CS−. This metric, in a given data set, is monotonically (inversely) related to measurement error (Bach et al., 2020; Bach & Melinscak, 2020; Bach, Tzovara, & Vunder, 2018).

## 2 METHOD

### 2.1 Participants

Two independent samples of healthy individuals were recruited from the general population. All participants confirmed that they had no history of neurological, psychiatric, or systemic medical conditions, and all had normal or corrected‐to‐normal vision. For Experiment 1 (data set code TFC1) we recorded data from 31 participants. One participant withdrew during acquisition, as they could not tolerate the US. Another participant did not return for the recall session, and one participant had incomplete data due to a technical failure. Thus, we included data of 28 participants into the analysis (7 males, 21–40 years, mean age ± *SD*: 27.25 ± 5.01 years). For Experiment 2 (data set code TFC2) we recorded data from 30 participants. One participant did not return for the recall session, and an additional participant was excluded due to malfunction of the US delivery. Therefore, we included data of 28 participants into the analysis (13 males, 20–39 years, mean age ± *SD*: 25.53 ± 4.54 years). The samples in Experiments 1 and 2 did not differ in age (*t*[54] = 1.34, *p* = .19, *d* = 0.36) or gender distribution (*χ* ^2^ = 1.95, *p* = .16). All participants gave written informed consent before the experiment. The study was conducted in accordance with the Declaration of Helsinki and approved by the governmental research ethics committee (Kantonale Ethikkomission Zürich KEK‐ZH‐2013‐0118).

### 2.2 Calibration approach and sample size

As in previous work, we followed a calibration strategy, which assumes that participants acquire and retain a CS‐US association, and aims to find the conditioned response quantification with the highest retrodictive validity, that is, effect size to differentiate CS+ and CS−. It has been shown that in such approach, high retrodictive validity reflects low measurement error (Bach et al., 2020). To determine sample size, our goal was to obtain a robust estimate of population retrodictive validity, rather than achieve high power in a null hypothesis significance test. As no formal framework is available for sample size calculation in this approach, sample size was heuristically based on previous methodological work. Post hoc, the relatively similar effect sizes observed in both experiments suggest a certain chance that robust effect size estimates were obtained.

### 2.3 Design and stimuli

Both experiments used the same design with an acquisition and a recall session. In the acquisition phase, participants were presented with 40 CS: 20 CS+ with 100% reinforcement, and 20 CS− that predicted the absence of the US. In the recall session 1 week later, US electrodes were attached, and participants were presented with the same CS 30 times: 15 CS+ and 15 CS−. None of the CS were reinforced, and a startle probe was delivered on each trial 13 s after CS offset (i.e., 2 s before the expected US delivery), both in CS+ and CS− trials. For both experimental phases, CS order was randomized for each participant, with the constraint that maximally three CS of the same type were presented consecutively.

CS were differently colored isoluminant triangles (yellow, RGB: 225, 224, 177; purple, RGB: 238, 194, 244) of ~4.1° visual angle, presented for 2 s at the center of an isoluminant gray (RGB: 175, 175, 175) computer screen. CS‐color relation was counterbalanced across participants. During the 15‐s trace interval, a white (RGB: 255, 255, 255) fixation cross of ~0.8° visual angle was presented at the center of the gray background screen. Attentiveness and stimulus recognition might be especially relevant for trace conditioning (Han et al., 2003), therefore participants were asked to indicate the color of the CS by pressing the left/right cursor keys during CS presentation on a standard computer keyboard. If participants gave the wrong response, the words “wrong key”, and in case of no response during CS presentation the words “no response”, were presented immediately after CS offset for 1 second.

During a 30‐s ITI, with a ±2‐s jitter, participants were instructed to perform a simple visual detection task, in order to reduce backward conditioning, as well as drowsiness due to the long ITIs (de Haan et al., 2018). We presented a stream of 13 white (RGB: 255, 255, 255) single digits with a red (RGB: 255, 0, 0) fixation cross embedded in the stream, at a rate of 1 Hz with a presentation time of 0.2 s. Participants were asked to respond with a keypress to the red cross. The onset of this task was randomized between 5–10 s after US offset.

After both experimental sessions, participants were asked to rate CS‐US contingency for both CS from 0 to 100. In Experiment 2 only, participants were additionally asked after the recall session to indicate the shock contingency for each CS in the acquisition session.

### 2.4 Settings and equipment

US were trains of 83 square electric pulses of 0.2 ms duration with a duty cycle of 1.67%, resulting in total US duration of approximately 1000 ms. Electric pulses were delivered on the participants' dominant forearm with a pin‐cathode/ring anode configuration with a constant current stimulator (Digitimer DS7A, Digitimer, Welwyn Garden City, UK). The stimulus was set to a perceived intensity of approximately 90% of a clearly painful stimulus. This pain threshold was estimated in two phases. In the first phase, the intensity was increased from an imperceptible to a painful level, thereby defining an upper limit for the second phase. In the second phase, participants were asked to rate the subjectively perceived intensity of 14 stimuli with different intensities. These ratings were linearly interpolated to estimate the intensity corresponding to 90% of the clearly painful stimulus. This estimate was then used in the experiment. This resulted in currents of 1.35–48.40 mA (mean ± *SD*: 4.84 ± 8.67 mA) in Experiment 1 and between 1.30–7.39 mA (mean ± *SD* = 4.05 ± 1.52 mA) in Experiment 2.

Startle probes were 20‐ms, instantaneous rise time, white noise sounds of 102 dB loudness, delivered binaurally with headphones (HD 202, Sennheiser, Wedemark–Wennebostel, Germany).

The experiment was presented on a Dell P2014h 20″ screen, set to an aspect ratio of 4:3 at 60 Hz, with a resolution of 1152 × 864 pixels. The experiments took place in a dark, soundproof chamber. Participants were positioned on a chin rest at 70 cm distance from the monitor and 47 cm from the eyetracker.

### 2.5 Psychophysiological recordings

We recorded the EMG from the orbicularis oculi muscle of participants' left eye with two 4 mm Ag/AgCl cup electrodes filled with high‐conductance gel. One was placed below the lower eyelid on the muscle in a line with the pupil in forward gaze, the other below the lateral canthus, at approximately 1–2 cm distance (Blumenthal et al., 2005). The electromyogram was amplified with a gain of 2000 and filtered with a band‐pass filter at 1 and 500 Hz (EMG100C, Biopac Systems). To measure skin conductance responses (SCR), disposable Ag/AgCl snap electrodes (EL507, Biopac Systems), filled with 0.5% NaCl (Hygge & Hugdahl, 1985) electrolyte gel (GEL101, Biopac Systems), were placed on the thenar/hypothenar of the non‐dominant hand. A ground electrode was additionally placed on the left elbow. SCR were measured with a 0.5 V constant voltage coupler/amplifier (EDA100C, Biopac Systems). To measure ECG, pre‐gelled disposable Ag/AgCl snap electrodes (01‐7500, TIGA‐MED) were placed on both wrists and above the right foot ankle. Lead I configuration was generated and amplified (ECG100C, Biopac Systems). Respiration was measured with a single‐belt cushion system (RSP100C, Biopac Systems). All signals were digitized (MP160, Biopac Systems) at 2000 Hz and recorded (Acknowledge, Biopac Systems).

Pupil diameter and gaze direction for both eyes were recorded with an EyeLink 1000 System (SR Research) at a sampling rate of 500 Hz. To calibrate gaze direction, we used the nine‐point protocol implemented in the EyeLink 1000 software.

### 2.6 Data analysis

Pre‐processing and analysis of psychophysiological data were performed using MATLAB (Version R2018a, Math‐Works) using procedures implemented in PsPM 4.1.1 (Psychophysiological modeling, http://pspm.sourceforge.net), a MATLAB toolbox for model‐based analysis of psychophysiological data (Bach & Friston, 2013; Bach, Tzovara, et al., 2018), and R 4.0.2 (R Core Team, 2020). For pre‐processing of pupil data only, we used PsPM 5.1.0 (bachlab.github.io/pspm).

#### 2.6.1 Data pre‐processing

##### EMG

EMG pre‐processing for quality control followed the procedure developed by Khemka et al. (2017). Data were filtered with a 4th order Butterworth filter with 50 and 470 Hz cut‐off frequencies. To remove mains noise, we used a 50 Hz notch filter. Data were rectified and then smoothed with 4th order Butterworth low‐pass filter with a time constant of 3 ms. Pre‐processed EMG was visually inspected. Three participants in Experiments 1, and 2 in Experiment 2, had no discernible average SEBR over all trials and were excluded from further EMG analysis.

##### Skin conductance

Data of some participants showed artifacts from the electric stimulation used as US. To account for this, the period from 0.2 s before US onset to 1.6 s after US onset was treated as missing values for all analyses. Two participants in Experiment 1, and 1 participant in Experiment 2, showed no visually discernible average skin conductance response to all US and were excluded from further SCR analyses.

##### Pupil size

We first converted from EyeLink 1000 system's arbitrary units to true diameter using the transform derived in Hayes and Petrov (2016). Data were then pre‐processed following the procedure by Kret and Sjak‐Shie (2019) as implemented in PsPM 5.1.0. In brief, this determines valid samples by range, speed, edge, trendline, and isolated sample filtering and then smoothes valid data by filtering, interpolation, and combination of data from both eyes. Finally data points for which gaze direction was outside ±5° visual angle were treated as missing data points. For time‐course analysis and visualization, data were interpolated and smoothed with a moving average of 1 s width. Trials with more than 50% missing data between CS onset to US onset were excluded from the analysis.

##### ECG

ECG pre‐processing followed Paulus et al. (2016) using the standard procedures implemented in PsPM. This uses a modified offline implementation of the Pan and Tompkins (Pan & Tompkins, 1985) real‐time QRS detection. Inter‐beat intervals (IBIs) were then assigned to the following heartbeat, while rejecting IBI values outside the interval 600–1200 ms (corresponding to 50–100 bpm). Heart period was linearly interpolated with 10 Hz sampling frequency and filtered with a fourth order bidirectional bandpass Butterworth filter (cut‐off frequencies: 0.015–0.5 Hz).

##### Respiration amplitude

Respiration data pre‐processing followed Bach et al. (2016) and Castegnetti et al. (2017) using the standard procedures implemented in PsPM. This algorithm detects the beginning of inspiration cycles and assigns cycle amplitude to the beginning of the next cycle. These data are then interpolated with 10 Hz sampling frequency. No additional filtering was implemented.

#### 2.6.2 Time course analysis

In a first analysis, we took a model‐free perspective to investigate the time course of conditioned responses in this paradigm. To confirm CS+/CS− differences, we used a cluster‐level random permutation test. We extracted individual trial data (0–17 s after CS onset), resampled at 10 Hz, for skin conductance, pupil size, heart period, and respiration amplitude. Missing data in SCR (due to US artifacts) and pupil size (due to loss of fixation) were linearly interpolated. Data were averaged over trials, separately for each condition per participant. We computed a paired *t*‐test for CS+ versus CS−, for each datapoint in the trial‐average time course, resulting in 170 *t*‐tests. We then permuted trial labels 10,000 times within each participant, each time averaging over trials and performing the same 170 *t*‐tests. We used a cluster‐level correction for multiple comparisons (Maris & Oostenveld, 2007), which controls the false positive rate for the statement that there is a condition difference anywhere within the time course. For illustration, we identified and visualized significant clusters within the trial‐average time course.

#### 2.6.3 Psychophysiological modeling

The second set of analyses  built on psychophysiological models, that is, forward models that specify how CS‐US association is expressed in behavior. These models were then inverted, which yields, separately for each trial or condition, an estimate to what extent CS‐US association is expressed in behavior; these were then compared between conditions.

##### 2.6.3.1 Startle eye‐blink response

For SEBR, two different methods were used. First, we used a general linear model, which quantifies for each trial an amplitude of the SEBR by linear regression of the EMG data onto canonical SEBR with variable onset (Khemka et al., 2017). This method used the pre‐processing strategy detailed above. Secondly, we employed a peak scoring method (Balderston et al., 2017) in the version used in (Khemka et al., 2017). To this end, we high‐pass filtered the raw EMG signal with a 4th order Butterworth filter at 30 Hz and applied a notch filter to remove 50 Hz mains noise. Filtered EMG data were rectified and smoothed using a 20‐ms moving average. The peak startle amplitude for each trial was then defined as the maximum EMG amplitude between 20 and 100 ms after startle sound onset as determined from recordings of the audio output.

For both analyses, each participant's amplitude estimate was normalized by dividing through the mean SEBR in this participant's CS− trials (Bach et al., 2019; Bach, Castegnetti, et al., 2018) to correct for differences in electrode impedance and muscle anatomy.

##### 2.6.3.2 Skin conductance responses

SCR pre‐processing and analysis extended the procedure developed by Staib et al. (2015). Data were filtered with a first order uni‐directional band‐pass Butterworth filter (0.0159–5 Hz) and downsampled to 10 Hz. We then employed two analysis methods. Firstly, we used the standard non‐linear model implemented in PsPM. This provides trial‐by‐trial estimates of sudomotor bursts, modeled as a Gaussian bump functions (Bach et al., 2010). We defined three bursts, two with constant latency after CS and US time points, and one with estimated latency (but fixed dispersion) during the trace interval, from 8 s after CS offset to 1 s before US onset. Each participant's amplitude estimate was normalized by dividing through the mean estimate in this participant's CS− trials (Bach et al., 2019; Bach, Castegnetti, et al., 2018). Secondly, we summarized SCR during the trace interval by computing the area under the curve (AUC) from CS onset to US timepoint using the PsPM module for spontaneous fluctuations (SF) (Bach et al., 2010). This analysis did not reveal any additional insights and is not included in the results.

##### 2.6.3.3 Pupil size responses

Previous work on delay fear conditioning with variable CS‐US interval has suggested that the anticipatory pupil size response (PSR) to CS+ is time‐locked to CS onset (Korn et al., 2017), but only delay fear conditioning procedures were tested. Here, we first estimated the anticipatory pupil response using PsPM's standard single‐trial GLM (Korn et al., 2017). Secondly, we built a specific response function on the pupil size time‐course during the acquisition session of Experiment 1. This model was then inverted again using the single‐trial GLM (Korn et al., 2017). For both inversions, we normalized each participant's amplitude estimates by dividing through the mean estimate in this participant's CS− trials.

##### 2.6.3.4 Heart period responses

In delay fear conditioning with variable CS‐US interval, the expression of fear‐conditioned bradycardia appeared to be time‐locked to the US (Castegnetti et al., 2016, 2017). However, US onset and CS offset co‐incided in these experiments, and so it is unclear how the model should be adapted for trace conditioning. Hence, we first inverted the US‐locked model, and secondly built a specific response function on the heart period time course during the recall session of Experiment 1. This model was then inverted using the standard GLM approach as in (Castegnetti et al., 2016).

##### 2.6.3.5 Respiration amplitude responses

For respiration amplitude responses (RAR), previous work provided some weak evidence that the expression of US anticipation may be time‐locked to the US (Castegnetti et al., 2017) although this was less clear than for heart period responses (HPR). Our time‐course analysis identified no appreciable CS+/CS‐ difference such that no new forward model could be identified. In an exploratory analysis, we used PsPM's standard condition‐wise GLM with early and late response functions (Castegnetti et al., 2017).

##### 2.6.3.6 Statistical analysis

Statistical Analysis was performed in R (www.r‐project.org), version 4.0.2. For the acquisition session, we averaged response estimates across all CS+ and CS− trials separately and computed a paired *t*‐test for the CS+/CS− difference. To account for potential extinction during the recall session, the time course of which is unknown, we averaged the first 1‐*n* CS+ and CS− trials, with n ranging from 1 to 15, and then performed 15 paired *t*‐tests for Experiment 1. The average with the highest effect size was then confirmed in Experiment 2. *T*‐test were performed using function t.test() of the package “stats” version 4.0.2, Cohen's *d* were estimated using cohensD() of package “lsr” version 0.5.

Because SEBR and SCR response estimates habituate over time, independently of extinction processes, and the sequence of CS+ and CS− is determined randomly, it might be more sensitive to analyze responses in a linear mixed effects model with trial number as predictor across conditions. To this end, we used linear mixed effects modeling as implemented in the R package “lme4” (version 1.1.23) with the function lmer(). For SEBR, we modeled habituation by an exponential decay. The decay parameter was fitted to data from all trials of Experiment 1, based on ordinary least squares, and then used for analysis of Experiments 1 and 2. For SCR, we used a linear decay. We tested for the effects of trial and condition (i.e., CS type), and their interaction. *F*‐statistics were determined using the anova() function from the package “car” version 3.0.9, *p*‐values with the pf() function of the package “stats” version 4.0.2. We fitted two models with different random effects structures, random intercept, or random effects for intercept and trial order, on data from Experiment 1, and selected the model with the smallest Akaike Information Criterion. For all LME model analysis, the model accounting for random effects for subject and trial was selected (formula: lmer(data ~ [1 + trial | subject] + trial * condition)). For Experiment 2, only the model selected in Experiment 1 was fitted. Effect sizes for LME were calculated using the R package “effectsize” version 0.5 with function eta_squared(),confidence intervals were computed using the package “MBESS” version 4.8.1, with the function ci.pvaf() both for SEBR and SCR. For robustness analysis of SEBR results, we additionally performed an ANOVA with trial as a predictor within condition, using the R function aov() of the stats package version 4.0.2, where effectsizes were calculated using “effectsize” version 0.5 with function eta_squared(), and cohens_f(), and confidence intervals were computed using the package “MBESS” version 4.8.1, with the function ci.pvaf().

## 3 RESULTS

### 3.1 Time‐course analysis

**FIGURE 1:**
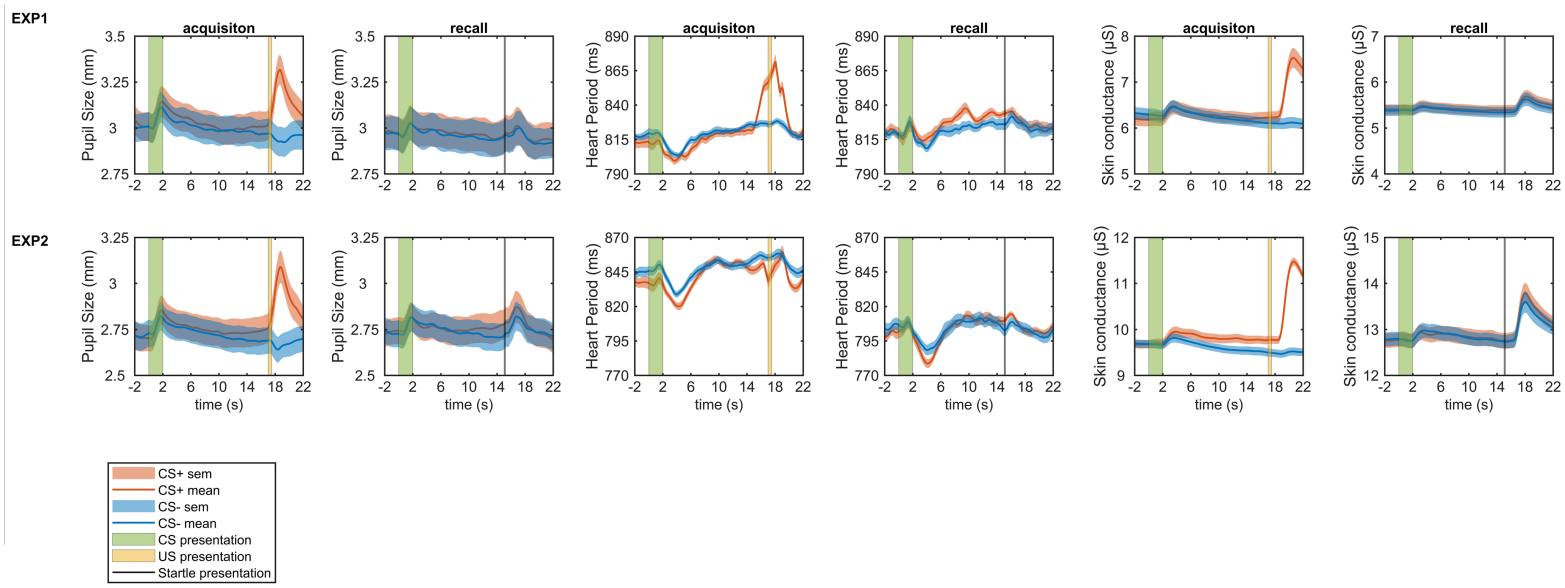
Averaged time course of pupil size, heart period data and skin conductance. Red line and shaded area: CS+ trials ± SEM, blue line and shaded area: CS− trials ± SEM, green section: CS presentation, yellow section: US presentation, black line: startle sound presentation.

We first analyzed conditioned responses in a model‐free way to ask how and when the anticipation of US might be expressed in psychophysiological signals. Notably, this analysis averaged over all trials such that it may be less suited to find conditioned responses with variable timing over trials or participants. Furthermore, we did not include SEBR in this analysis as they are always locked to the startle probe. Although condition differences were visible in pupil size and heart period (Figure 1), none of these were significant after correction for multiple comparisons across data points. No condition differences were found for skin conductance and respiration amplitude in Experiment 1.

### 3.2 Analysis of response estimates

Next, we built on psychophysiological models that specify how US expectation is expressed in conditioned responses, and derive, for each trial or condition, an estimate of US expectation.

#### 3.2.1 Startle eye‐blink responses

**FIGURE 2:**
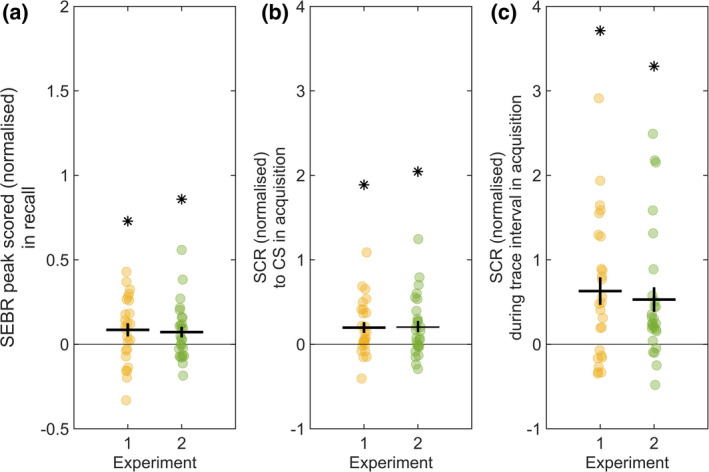
Scatterplot of CS+/CS− differences in SEBR and SCR. (a). Normalized SEBR (peak scored) difference between CS+/CS− in the recall session, averaged over all trials. (b). Normalized SCR difference between CS+/CS− at CS presentation in the acquisition session. (c). Normalized SCR difference between CS+/CS− during the trace interval in the acquisition session. Average values are depicted with a horizontal line, SEM with a vertical line, scatterplots show individual values, asterisks denote significant difference (*p* < .05) from 0.

First, we separately averaged the first 1–15 CS+ and CS− trials for each participant and performed 15 paired *t*‐tests to compare the conditions (see supplementary information). For the GLM estimates, there were no significant condition differences in either of the two experiments. For the peak‐scoring estimates in Experiment 1, the CS+/CS− difference in SEBR was most pronounced when averaging over all 15 trials (*t*[24] = 2.19, *p* = .038, Cohen's *d* = 0.44). This result was confirmed in experiment 2 (*t*[25] = 2.25, *p* = .033, *d* = 0.44). Effects sizes from all comparisons are summarized in Table 1, and results are illustrated in Figure *2*.

**TABLE 1:** SEBR results, averaged over all trials of the recall session, for Experiments 1–2 for peak scoring and model inversion analysis

	Method	Cohen's *d*	Hedge's *g*
Experiment 1	Peak scoring	0.44	0.42
	GLM	0.35	0.34
Experiment 2	Peak scoring	0.44	0.43
	GLM	0.37	0.36

For GLM estimates, a linear mixed effects model with trial as a factor across conditions and random effects accounting for trial and subject intercept revealed response habituation over trials (main effect trial: *F*[1, 722] = 47.45, *p* < .001, *η* ^2^ = 0.66) and larger responses in CS+ compared to CS− trials (main effect condition: *F*[1, 722] = 4.68, *p* = .031, *η* ^2^ = 0.01) with no interaction (*F*[1, 722] = 1.97, *p* = .161, *η* ^2^ < 0.01) in Experiment 1. Both the effect of trial (*F*[1, 751] = 28.80, *p* < .001, *η* ^2^ = 0.42) and the effect of condition (*F*[1, 751] = 8.74, *p* = .003, *η* ^2^ < 0.01) were confirmed in Experiment 2, the interaction (*F*[1, 751] = 0.08, *p* = .772, *η* ^2^ < 0.01) was again not significant.

For peak‐scoring estimates, the LME model showed response habituation over trials (main effect trial: *F*[1, 722] = 61.84, *p* < .001, *η* ^2^ = 0.72) and larger responses in CS+ compared to CS− trials (main effect condition: *F*[1, 722] = 5.48, *p* = 0.020, *η* ^2^ = 0.01) with no interaction (*F*[1, 722] = 1.92, *p* = 0.167, *η* ^2^ < 0.01) in experiment 1. This was replicated in experiment 2 for trial (*F*[1, 751] = 80.03, *p* < 0.001, *η* ^2^ = 0.76) and condition (*F*[1, 751] = 6.04, *p* = 0.014, *η* ^2^ = 0.01), while the interaction was again not significant (*F*[1, 751] = 1.08, *p* = 0.30, *η* ^2^ < 0.01). To verify robustness of the LME model results, we also conducted an ANOVA on the peak‐scoring estimates, with trial number within conditions as factor. This confirmed significant main effects of CS (*F*[1690] = 4.77, *p* = .029, *η* ^2^ = 0.01, Cohen's *f* = 0.08) and trial number (*F*[29,690] = 7.43, *p* < .001, *η* ^2^ = 0.23, *f* = 0.56) with no interaction (*F*[29, 690] = 0.80, *p* = .760, *η* ^2^ = 0.02, *f* = 0.18). This was further confirmed in experiment 2 (CS: *F*(1, 720) = 7.20, *p* = .008, *η* ^2^ = 0.01, *f* = 0.10, trial number: *F*(29, 720) = 10.05, *p* < .001, *η* ^2^ = 0.28, *f* = 0.64; interaction: *F*(29, 720) = 0.95, *p* = .506, *η* ^2^ = 0.03, *f* = 0.20).

#### 3.2.2 Skin conductance responses

Averaged across the entire acquisition session, estimated SCR amplitudes in Experiment 1 were higher in CS+ compared to CS− trials, both for the timepoint of CS presentation (*t*[25] = 3.14, *p* = .004, *d* = 0.22) and during the trace interval (*t*[25] = 3.87, *p* = .001, *d* = 0.43) respectively. This result was confirmed in Experiment 2 for the timepoint of CS presentation (*t*[26] = 3.12, *p* = .004, *d* = 0.20) and the trace interval (*t*[26] = 3.62, *p* = .001, *d* = 0.32).

For the recall phase in Experiment 1, paired t‐tests revealed no significant (*p* < .050) difference between CS+/CS− trials during CS presentation or the trace interval, irrespective of the number of trials included into the analysis (see supplementary information). For the time point of US presentation, CS− amplitudes were significantly higher than CS+ amplitudes when averaged over 1–11, 1–12 or 1–13 trials, but not when all trials where included, with the largest effect size for 12 trials. However, this result was not confirmed in Experiment 2, nor did any other significant condition difference emerge in Experiment 2.

Linear mixed‐effects modeling of the normalized data during the recall phase revealed significant habituation (*main effect trial*: *F*[1, 809] = 35.28, *p* < .001, η^2^ = 0.57), *larger CS+ than CS− responses* (*main effect condition*: *F*[1, 809] = 5.53, *p* = .019, *η* ^2^ = 0.02) *and an interaction* (*F*[1, 809] = 10.94, *p* < .001, *η* ^2^ = 0.01) during the trace interval. However, while the effect of trial was replicated (*F*[1, 780] = 21.01, *p* < .001, *η* ^2^ = 0.45*)* the effect of condition was not confirmed in experiment 2 (*F*[1, 780] = 0.190, *p* = .17, *η* ^2^ < 0.01), nor was the interaction (*F*[1, 780] = 1.55, *p* = 0.21, *η* ^2^ < 0.01*)*. A combined analysis for both datasets again confirmed the effect of trial (*F*[1, 1563] = 42.84, *p* < .001, *η* ^2^ = 0.45) but not of condition (*F*[1, 1563] = 3.80, *p* = 0.051, *η* ^2^ < 0.01), or interaction (*F*[1, 1563] = 3.10, *p* = .078, *η* ^2^ < .01).

#### 3.2.3 Pupil dilation

Estimated CS‐locked anticipatory PSR, averaged across the entire acquisition session, show a significant difference between CS+/CS− trials in Experiment 1 (*t*[27] = 3.10, *p* = .004, *d* = 0.59). This result was not confirmed in Experiment 2 (*t*[27] = 1.34, *p* = .191, *d* = 0.25). None of the paired *t*‐tests for the recall session between CS+/CS− trials in either experiment reached significance level (*p* < .050), irrespective of how many trials were included into the analysis (see supplementary information). Similarly, in Experiment 2 no significant difference between CS+/CS− trials was observable for the recall session.

Estimated pupil dilation, fitted to time course of Experiment 1, showed larger response to CS+ than CS− trials in the acquisition session (*t*[27] = 8.20, *p* < .001, *d* = 1.55). This results was replicated (without re‐fitting the pupil response) in Experiment 2 (*t*[27] = 5.94, *p* < .001, *d* = 1.12). Paired sample *t*‐test for the recall session were not significant, irrespective of how many trials were included, neither for Experiment 1 nor 2.

#### 3.2.4 Heart period

Estimated US‐locked bradycardia amplitudes were not significantly different between CS+ and CS− in either experiment. Based on the observed heart period time courses in the recall session of Experiment 1, we built a response function and estimated responses in Experiment 2. This analysis did not reveal any significant results.

#### 3.2.5 Respiration amplitude

We found no CS+/CS− differences in the acquisition phase in either experiment. Smaller respiration amplitude responses for CS+ versus CS− trials during recall in Experiment 1 (*t*[27] = 3.55, *p* = .001, *d* = 0.67) were not confirmed in Experiment 2 (*t*[27] = 0.30, *p* = .768, *d* = 0.06).

### 3.3 Declarative memory and task performance

**FIGURE 3:**
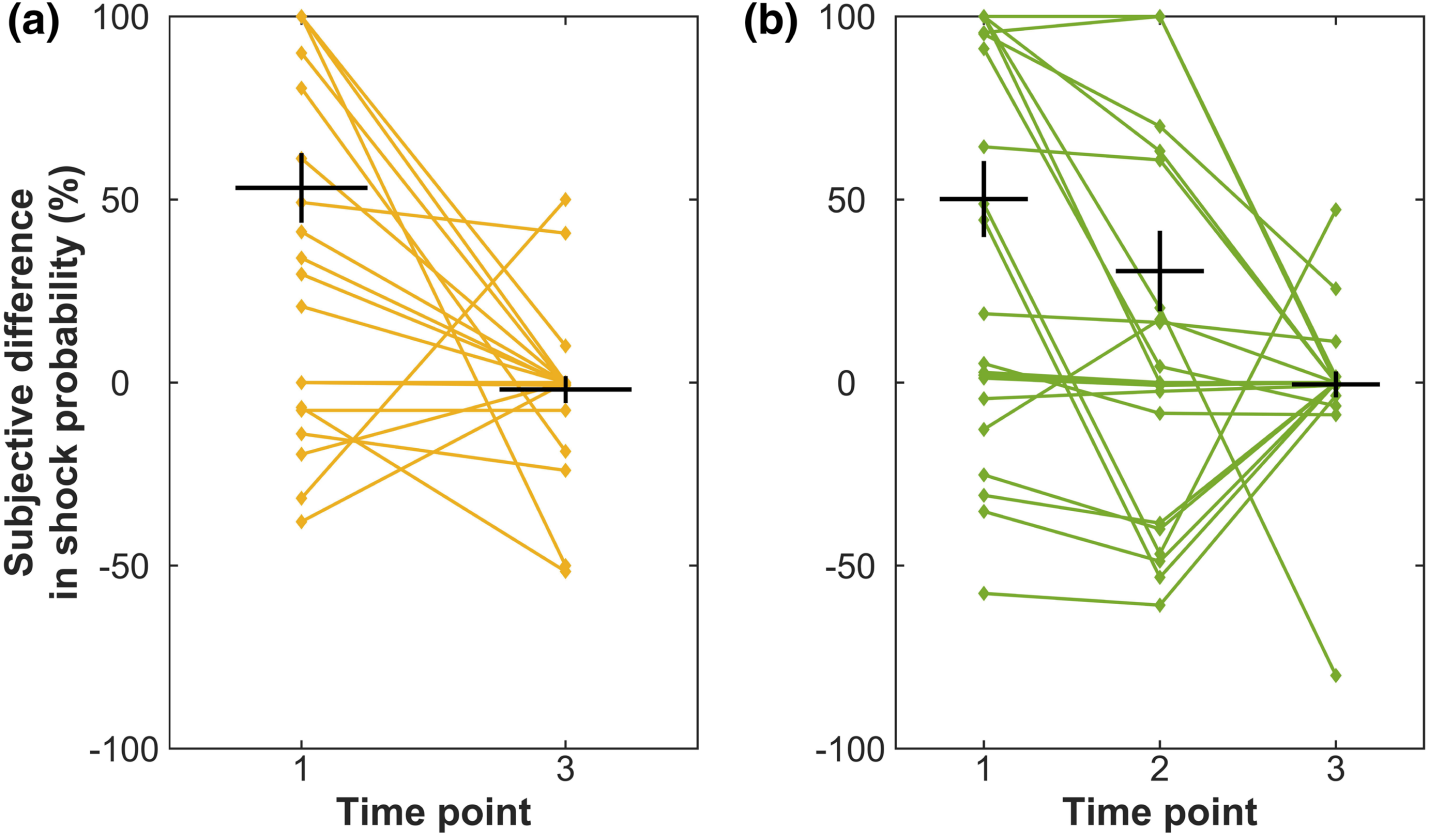
Contingency ratings, displayed as CS+/CS‐ differences. CS+/CS− difference in actual contingency is 100% in acquisition and 0% in recall session. (a) Experiment 1, individual ratings depicted in yellow. (b) Experiment 2. Individual ratings depicted in green. Mean values are marked with a horizontal black line, SEM is depicted with a vertical line. Timepoints: 1—directly after the acquisition phase, 2—acquisition contingency as remembered after the recall session, 3—recall session.

Participants learned the CS+/CS− contingency differences (paired *t*‐tests, *p* < .001), remembered them until after the recall session (paired *t*‐test, *p* = .010, for Exp. 2), and learned the new contingency for CS+ in the recall session (paired *t*‐test, *p* < .001, for acquisition—recall), although their numerical estimates substantially deviated from the correct ones (Table 2, Figure 3). Performance and accuracy in the incidental CS identification task were above 95% in most metrics (Table 3). Hit rate in the ITI task was above 90% and the false alarm rate was below 4% in all metrics (Table 4).

**TABLE 2:** Objective shock presentation and subjective contingency ratings for shock and CS‐US association in Experiments 1 and 2. Participants were asked to indicate how likely they thought each CS was followed by a US. In Experiment 2, participants were additionally asked after the recall session to remember how likely CS was followed by US in the acquisition session

	Session	Experiment	US contingency (%)	Mean contingency rating (%) (±*SD*)
CS+	Acquisition	1	100	78.51 ± 24.11
		2	100	76.79 ± 26.74
	Memory	2	100	65.07 ± 33.11
	Recall	1	0	11.89 ± 22.38
		2	0	10.49 ± 21.38
CS−	Acquisition	1	0	25.34 ± 27.84
		2	0	26.66 ± 30.95
	Memory	2	0	34.61 ± 32.49
	Recall	1	0	13.79 ± 27.22
		2	0	10.99 ± 22.46

**TABLE 3:** Performance (percentage of trials with a response) and accuracy (percentage of correct responses in all trials) for the incidental CS identification task in the two experiments

	Mean (±*SD*)	
	Performance (%)	Accuracy (%)
Experiment 1		
Acquisition	97.05 ± 5.05	94.11 ± 8.37
Recall	98.81 ± 1.63	96.79 ± 4.84
Experiment 2		
Acquisition	98.21 ± 2.88	96.61 ± 4.04
Recall	98.10 ± 6.38	96.67 ± 7.37

**TABLE 4:** Hit rate (percentage of trials with a response to the target stimuli) and false alarm rate (percentage of trials with a response to non‐target stimuli) for the ITI task

	Mean (±*SD*)	
	Hit rate (%)	False alarms (%)
Experiment 1		
Acquisition	91.16 ± 25.28	2.14 ± 4.23
Recall	98.33 ± 3.80	1.67 ± 4.11
Experiment 2		
Acquisition	96.96 ± 4.05	2.77 ± 3.49
Recall	93.45 ± 17.79	3.69 ± 5.62

## 4 DISCUSSION

Trace fear conditioning is an important model of realistic biological and clinical aversive learning scenarios, where predictors and outcomes are separated in time. In rodent studies, trace intervals longer than 10 s clearly involve hippocampal learning. The best strategy for quantifying learning and memory at such long trace intervals in humans is currently unclear. In this study, we conducted two independent trace fear conditioning experiments with a 15‐s trace interval, to establish a method for quantification of learning and of memory retention. We included several data types in which conditioned responses are commonly observed in delay fear conditioning paradigms. Our exploration‐confirmation strategy allowed us to freely investigate many possible analysis algorithms in Experiment 1 and confirm the most sensitive ones in Experiment 2. We used retrodictive validity as a metric to assess measurement error (Bach et al., 2020; Bach & Melinscak, 2020; Bach, Tzovara, & Vunder, 2018).

The first finding is that among psychophysiological measures, only SEBR differentiated CS+ and CS− trials in a recall test 1 week after acquisition. As the most sensitive analysis, a previously proposed peak‐scoring protocol emerged (Balderston et al., 2017), which was superior to GLM‐based amplitude estimates (Khemka et al., 2017). These results replicated across both experiments and were robust in *t*‐tests, as well as linear mixed effects models and repeated‐measures ANOVA accounting for SEBR habituation. We found no evidence of SEBR extinction over 30 trials in the recall session. Effect size to distinguish CS+/CS− was *d* = 0.44, which would require 34 participants to achieve 80% power of demonstrating this difference in a one‐tailed *t*‐test at an alpha level of .05, and 514 participants to demonstrate an at least 50% reduction in fear memory in intervention vs. placebo trial.

During the acquisition session, SCR and PSR were the only psychophysiological measures that differentiated CS+/CS− trials. Our SCR model encompassed a response to the CS and one during the trace interval; both responses differentiated CS+/CS−, with the trace interval response being more sensitive. This result was replicated across both experiments. Additionally, our PSR model fitted on time‐course data of Experiment 1 revealed significant CS+/CS− differentiation for Experiment 2. We were unable to establish fear retention in SCR and PSR. Participants recalled a robust if imprecise declarative memory representation of the CS‐US association. Memory for the initial CS‐US association was remembered until after the recall session when appropriately prompted.

It has already been established that learning CS‐US associations in trace fear conditioning with shorter intervals can be measured with psychophysiological measures (Büchel et al., 1999; Haritha et al., 2013). The challenge in the present study was the long 15‐s trace interval, and the recall test 1 week after acquisition. To our knowledge, aversive memory quantification in such paradigm has not yet been probed.

Regarding our SEBR finding, it is typically assumed that CS‐US association is extinguished during a recall test without reinforcement, and hence memory would be best quantified during the first few trials of recall. This is reflected in our previous results in delay fear conditioning, where SEBR averaged over the first 3–5 CS+ and CS− trials of the recall session provided the best measure of US memory (Khemka et al., 2017), both for PsPM model inversion and peak‐scoring. In the present study, fear retention was only measurable when averaged over all trials of the recall session. While our main results favor a peak‐scoring strategy, the same pattern was found for GLM inversion, where highest retrodictive validity—even if non‐significant—is achieved when including all trials of the recall session. This is in keeping with the lack of evidence for extinction in the current study. This might indicate that trace fear memory is harder to extinguish, as previously suggested (Ewald et al., 2014). This would be of general importance both to understand the basic neurobiology of aversive learning in biological scenarios, and perhaps even more so in clinical intervention research. Future studies might include a larger number of trials in the recall session to investigate the time course of extinction.

A priori, a drawback of the exploration‐confirmation approach with relatively modest sample size poses a certain risk that an analysis overfitted to the exploratory data set does not generalize to the confirmatory data set, while a less specific analysis might be sensitive to both experiments. This is only a concern for those results that did not generalize from Experiment 1 to Experiment 2 and is addressed by repeating the exploration procedure in the confirmation data set, and by analyzing the combined data sets, which did not reveal any additional insights here.

We based the choice of trace interval duration on previous rodent work. A drawback in comparing rodent and human fear conditioning is that rodent work typically uses single‐cue protocols, whereas differential conditioning is common in human work (Haaker et al., 2019; Lonsdorf et al., 2017) and was also used in the present study. Differential conditioning might involve additional learning processes related to the CS‐. In rodents, this additional safety learning is associated with activation in the bed nucleus stria terminal (BNST) (Foilb et al., 2021) and ventrolateral orbitofrontal cortex (vlOFC) (Sarlitto et al., 2018). In humans, the ventromedial prefrontal cortex (vmPFC) has been found to play an important role (Savage et al., 2020). It remains to be shown whether and to what extent human hippocampus is required for delay and trace fear conditioning, as the literature is sparse in this respect (Bechara et al., 1995).

While we investigated multiple measures and analysis methods, the trace conditioning paradigm itself might also be varied to improve the quantification of trace fear memory. For example, one could vary the timepoint of startle sound presentation, assess a potential impact of its perceived aversiveness, remove the task during ITI, or add startle sounds during acquisition to improve comparability between sessions. Furthermore, research into trace fear conditioning memory might help to elucidate these open questions.

To summarize, we identify suitable measures for trace fear conditioning: SEBR during recall, and SCR/PSR during acquisition. We hope that these results will be useful for substantive research in the field of aversive memory modification.

## AUTHOR CONTRIBUTIONS

**Jelena M. Wehrli:** Conceptualization; data curation; formal analysis; investigation; project administration; visualization; writing – original draft. **Yanfang Xia:** Conceptualization; data curation; investigation; methodology. **Samuel Gerster:** Resources; software. **Dominik R Bach:** Conceptualization; formal analysis; funding acquisition; methodology; project administration; resources; software; supervision; writing – original draft.

## FUNDING INFORMATION

This work was supported by the Clinical Research Priority Program of the University of Zurich for the CRPP “Synapse & Trauma”. DRB is supported by funding from the European Research Council (ERC) under the European Union's Horizon 2020 research and innovation programme (Grant agreement No. ERC‐2018 CoG‐816,564 ActionContraThreat). The Wellcome Centre for Human Neuroimaging is supported by core funding from the Wellcome (203,147/Z/16/Z)

## CONFLICT OF INTEREST

The authors have no known conflict of interest to disclose.

## Supporting information


**TABLE S1** Experiment 1: recall SEBR GLM modelled paired *t*‐test, not corrected for multiple comparisons
**TABLE S2** Experiment 2: recall SEBR GLM modelled paired *t*‐test, not corrected for multiple comparisons
**TABLE S3** Experiment 1: recall SEBR peak scored paired *t*‐test, not corrected for multiple comparisons
**TABLE S4** Experiment 2: recall SEBR peak scored paired *t*‐test, not corrected for multiple comparisons
**TABLE S5** Experiment 1: recall SCR DCM modelled paired *t*‐test, not corrected for multiple comparisons
**TABLE S6** Experiment 2: recall SCR DCM modelled paired *t*‐test, not corrected for multiple comparisons
**TABLE S7** Experiment 1: recall PSR standard RF, paired *t*‐test, not corrected for multiple comparisons
**TABLE S8** Experiment 2: recall PSR standard RF, paired *t*‐test, not corrected for multiple comparisons
**TABLE S9** Experiment 1: recall PSR fitted RF, paired *t*‐test, not corrected for multiple comparisons
**TABLE S10** Experiment 2: recall PSR fitted RF, paired *t*‐test, not corrected for multiple comparisons
**TABLE S11** Experiment 1: recall SF, paired *t*‐test, not corrected for multiple comparisons
**TABLE S12** Experiment 2: recall SF, paired *t*‐test, not corrected for multiple comparisons
**TABLE S13** Acquisition paired t‐test CS+/CS−, not corrected for multiple comparisons
**TABLE S14** Paired t‐test CS+/CS−, not corrected for multiple comparisons
**TABLE S15** Experiment 1: SCR DCM estimated response peak during trace interval in s (from start of trial)
**TABLE S16** Experiment 2: SCR DCM estimated response peak during trace interval in s (from start of trial)
**TABLE S17** Experiment 1: SCR LME in recall
**TABLE S18** Experiment 2: SCR LME in recall
**TABLE S19** Experiment 1 and 2 combined: SCR LME in recall
**TABLE S20** Experiment 1: SEBR GLM LME in recall
**TABLE S21** Experiment 2: SEBR GLM LME in recall
**TABLE S22** Experiment 1: SEBR peak scoring LME in recall
**TABLE S23** Experiment 2: SEBR peak scoring LME in recall
**TABLE S24** Experiment 1: SEBR peak scoring ANOVA in recall
**TABLE S25** Experiment 2: SEBR peak scoring ANOVA in recall

## Data Availability

Analysis code is available from OSF (https://osf.io/wbkfj/), all anonymized data sets are available on www.zenodo.org (Experiment 1: https://doi.org/10.5281/zenodo.6024202, Experiment 2: https://doi.org/10.5281/zenodo.6024245).
