# Supplementary material for: Measuring human trace fear conditioning
Source: Psychophysiology. 2022 Jun 8;59(12):e14119. doi: 10.1111/psyp.14119 (PMC9787976; doi:10.1111/psyp.14119)
Supplement: Supplementary file 1 — TABLE S1 Experiment 1: recall SEBR GLM modelled paired t‐test, not corrected for multiple comparisons TABLE S2 Experiment 2: recall SEBR GLM modelled paired t‐test, not corrected for multiple comparisons TABLE S3 Experiment 1: recall SEBR peak scored paired t‐test, not corrected for multiple comparisons TABLE S4 Experiment 2: recall SEBR peak scored paired t‐test, not corrected for multiple comparisons TABLE S5 Experiment 1: recall SCR DCM modelled paired t‐test, not corrected for multiple comparisons TABLE S6 Experiment 2: recall SCR DCM modelled paired t‐test, not corrected for multiple comparisons TABLE S7 Experiment 1: recall PSR standard RF, paired t‐test, not corrected for multiple comparisons TABLE S8 Experiment 2: recall PSR standard RF, paired t‐test, not corrected for multiple comparisons TABLE S9 Experiment 1: recall PSR fitted RF, paired t‐test, not corrected for multiple comparisons TABLE S10 Experiment 2: recall PSR fitted RF, paired t‐test, not corrected for multiple comparisons TABLE S11 Experiment 1: recall SF, paired t‐test, not corrected for multiple comparisons TABLE S12 Experiment 2: recall SF, paired t‐test, not corrected for multiple comparisons TABLE S13 Acquisition paired t‐test CS+/CS−, not corrected for multiple comparisons TABLE S14 Paired t‐test CS+/CS−, not corrected for multiple comparisons TABLE S15 Experiment 1: SCR DCM estimated response peak during trace interval in s (from start of trial) TABLE S16 Experiment 2: SCR DCM estimated response peak during trace interval in s (from start of trial) TABLE S17 Experiment 1: SCR LME in recall TABLE S18 Experiment 2: SCR LME in recall TABLE S19 Experiment 1 and 2 combined: SCR LME in recall TABLE S20 Experiment 1: SEBR GLM LME in recall TABLE S21 Experiment 2: SEBR GLM LME in recall TABLE S22 Experiment 1: SEBR peak scoring LME in recall TABLE S23 Experiment 2: SEBR peak scoring LME in recall TABLE S24 Experiment 1: SEBR peak scoring ANOVA in recall TABLE S25 Experiment 2: SEBR peak scoring [file PSYP-59-e14119-s001.pdf]

Table S1

Experiment 1: recall SEBR GLM modelled paired t-test, not corrected for multiple comparisons

							Mean (± SD)	
**trials**	**t-statistic**	**p**	**df**	**95% CI**	**cohen's d**	**hedge's g**	**CS+**	**CS-**
1-1	0.58	0.57	24	[-0.28, 0.50]	0.12	0.11	1.58 ± 0.78	1.47 ± 0.60
1-2	-0.22	0.83	24	[-0.31, 0.26]	0.04	0.04	1.48 ± 0.57	1.51 ± 0.45
1-3	-0.32	0.75	24	[-0.23, 0.17]	0.06	0.06	1.39 ± 0.43	1.42 ± 0.35
1-4	-0.28	0.78	24	[-0.16, 0.12]	0.06	0.05	1.34 ± 0.32	1.36 ± 0.30
1-5	0.06	0.95	24	[-0.12, 0.13]	0.01	0.01	1.32 ± 0.28	1.31 ± 0.23
1-6	0.71	0.49	24	[-0.07, 0.14]	0.14	0.14	1.29 ± 0.28	1.25 ± 0.19
1-7	0.24	0.81	24	[-0.08, 0.11]	0.05	0.05	1.23 ± 0.24	1.22 ± 0.16
1-8	-0.05	0.96	24	[-0.09, 0.08]	0.01	0.01	1.18 ± 0.21	1.18 ± 0.13
1-9	0.30	0.77	24	[-0.07, 0.09]	0.06	0.06	1.15 ± 0.19	1.14 ± 0.11
1-10	0.83	0.42	24	[-0.04, 0.10]	0.17	0.16	1.14 ± 0.16	1.11 ± 0.08
1-11	1.17	0.25	24	[-0.03, 0.10]	0.23	0.23	1.12 ± 0.16	1.08 ± 0.06
1-12	1.23	0.23	24	[-0.02, 0.10]	0.25	0.24	1.10 ± 0.15	1.06 ± 0.05
1-13	1.46	0.16	24	[-0.02, 0.11]	0.29	0.28	1.09 ± 0.17	1.04 ± 0.04
1-14	1.59	0.13	24	[-0.02, 0.12]	0.32	0.31	1.07 ± 0.16	1.02 ± 0.02
1-15	1.74	0.09	24	[-0.01, 0.12]	0.35	0.34	1.05 ± 0.15	1.00 ± 0.00

Table S2

Experiment 2: recall SEBR GLM modelled paired t-test, not corrected for multiple comparisons

							Mean (± SD)	
**trials**	**t-statistic**	**p**	**df**	**95% CI**	**cohen's d**	**hedge's g**	**CS+**	**CS-**
1-1	0.97	0.34	25	[-0.11, 0.30]	0.19	0.19	1.47 ± 0.53	1.37 ± 0.45
1-2	0.77	0.45	25	[-0.08, 0.18]	0.15	0.15	1.38 ± 0.37	1.33 ± 0.25
1-3	0.08	0.94	25	[-0.14, 0.15]	0.02	0.02	1.32 ± 0.28	1.32 ± 0.22
1-4	-0.23	0.82	25	[-0.12, 0.09]	0.04	0.04	1.25 ± 0.23	1.26 ± 0.15
1-5	0.09	0.93	25	[-0.09, 0.10]	0.02	0.02	1.23 ± 0.22	1.22 ± 0.13
1-6	0.19	0.85	25	[-0.08, 0.10]	0.04	0.04	1.23 ± 0.21	1.22 ± 0.14
1-7	0.42	0.68	25	[-0.07, 0.10]	0.08	0.08	1.21 ± 0.21	1.19 ± 0.11
1-8	0.47	0.64	25	[-0.07, 0.10]	0.09	0.09	1.18 ± 0.21	1.16 ± 0.09
1-9	0.63	0.53	25	[-0.06, 0.11]	0.12	0.12	1.15 ± 0.20	1.12 ± 0.07
1-10	0.93	0.36	25	[-0.04, 0.11]	0.18	0.18	1.12 ± 0.18	1.09 ± 0.06
1-11	1.23	0.23	25	[-0.03, 0.12]	0.24	0.23	1.11 ± 0.17	1.07 ± 0.05
1-12	1.29	0.21	25	[-0.03, 0.11]	0.25	0.24	1.09 ± 0.17	1.05 ± 0.04
1-13	1.66	0.11	25	[-0.01, 0.13]	0.32	0.31	1.09 ± 0.18	1.03 ± 0.02
1-14	1.69	0.10	25	[-0.01, 0.12]	0.33	0.32	1.07 ± 0.16	1.02 ± 0.02
1-15	1.87	0.07	25	[-0.01, 0.12]	0.37	0.36	1.06 ± 0.16	1.00 ± 0.00

Table S3

Experiment 1: recall SEBR peak scored paired t-test, not corrected for multiple comparisons

							Mean (± SD)	
**trials**	**t-statistic**	**p**	**df**	**95% CI**	**cohen's d**	**hedge's g**	**CS+**	**CS-**
1-1	0.56	0.58	24	[-0.40, 0.69]	0.11	0.11	1.80 ± 1.07	1.65 ± 0.74
1-2	-0.42	0.68	24	[-0.51, 0.33]	0.08	0.08	1.68 ± 0.76	1.77 ± 0.75
1-3	-0.48	0.63	24	[-0.37, 0.23]	0.10	0.09	1.54 ± 0.57	1.61 ± 0.57
1-4	-0.29	0.77	24	[-0.25, 0.19]	0.06	0.06	1.50 ± 0.46	1.53 ± 0.46
1-5	-0.08	0.94	24	[-0.21, 0.19]	0.02	0.02	1.47 ± 0.42	1.48 ± 0.34
1-6	0.53	0.60	24	[-0.12, 0.20]	0.11	0.10	1.42 ± 0.37	1.38 ± 0.28
1-7	0.09	0.93	24	[-0.14, 0.15]	0.02	0.02	1.33 ± 0.33	1.32 ± 0.23
1-8	-0.04	0.97	24	[-0.13, 0.13]	0.01	0.01	1.26 ± 0.30	1.27 ± 0.18
1-9	0.33	0.74	24	[-0.09, 0.13]	0.07	0.06	1.23 ± 0.27	1.21 ± 0.15
1-10	0.83	0.41	24	[-0.06, 0.14]	0.17	0.16	1.20 ± 0.23	1.16 ± 0.11
1-11	1.31	0.20	24	[-0.03, 0.14]	0.26	0.25	1.18 ± 0.22	1.13 ± 0.08
1-12	1.56	0.13	24	[-0.02, 0.14]	0.31	0.30	1.15 ± 0.22	1.09 ± 0.06
1-13	1.87	0.073	24	[-0.01, 0.16]	0.37	0.36	1.13 ± 0.22	1.06 ± 0.05
1-14	2.02	0.055	24	[0.00, 0.17]	0.40	0.39	1.11 ± 0.21	1.03 ± 0.02
1-15	2.19	0.038*	24	[0.01, 0.17]	0.44	0.42	1.09 ± 0.19	1.00 ± 0.00

Table S4

Experiment 2: recall SEBR peak scored paired t-test, not corrected for multiple comparisons

							Mean (± SD)	
**trials**	**t-statistic**	**p**	**df**	**95% CI**	**cohen's d**	**hedge's g**	**CS+**	**CS-**
1-1	-0.42	0.68	25	[-0.31, 0.20]	0.08	0.08	1.55 ± 0.59	1.61 ± 0.77
1-2	-0.25	0.81	25	[-0.16, 0.13]	0.05	0.05	1.47 ± 0.42	1.49 ± 0.37
1-3	0.28	0.78	25	[-0.12, 0.16]	0.05	0.05	1.42 ± 0.32	1.41 ± 0.27
1-4	0.33	0.75	25	[-0.08, 0.12]	0.06	0.06	1.36 ± 0.29	1.34 ± 0.19
1-5	0.89	0.38	25	[-0.05, 0.14]	0.17	0.17	1.33 ± 0.30	1.29 ± 0.16
1-6	0.98	0.33	25	[-0.05, 0.13]	0.19	0.19	1.32 ± 0.29	1.28 ± 0.18
1-7	0.92	0.37	25	[-0.06, 0.15]	0.18	0.17	1.30 ± 0.30	1.26 ± 0.17
1-8	0.90	0.38	25	[-0.06, 0.14]	0.18	0.17	1.26 ± 0.28	1.21 ± 0.14
1-9	0.99	0.33	25	[-0.05, 0.14]	0.19	0.19	1.22 ± 0.27	1.17 ± 0.11
1-10	1.21	0.24	25	[-0.04, 0.14]	0.24	0.23	1.18 ± 0.24	1.13 ± 0.09
1-11	1.55	0.13	25	[-0.02, 0.15]	0.30	0.29	1.17 ± 0.23	1.10 ± 0.07
1-12	1.71	0.10	25	[-0.01, 0.15]	0.34	0.32	1.13 ± 0.21	1.06 ± 0.05
1-13	2.06	0.050	25	[0.00, 0.15]	0.40	0.39	1.11 ± 0.19	1.04 ± 0.03
1-14	1.96	0.061	25	[0.00, 0.14]	0.38	0.37	1.08 ± 0.17	1.02 ± 0.03
1-15	2.25	0.033*	25	[0.01, 0.14]	0.44	0.43	1.07 ± 0.16	1.00 ± 0.00

Table S5

Experiment 1: recall SCR DCM modelled paired t-test, not corrected for multiple comparisons

								Mean (± SD)	
	**trials**	**t-statistic**	**p**	**df**	**95% CI**	**cohen's d**	**hedge's g**	**CS+**	**CS-**
to CS presentation	1-1	-0.39	0.70	25	[-1.00, 0.68]	0.08	0.07	1.05 ± 1.37	1.21 ± 1.06
	1-2	-0.57	0.57	25	[-0.58, 0.33]	0.11	0.11	1.40 ± 1.01	1.53 ± 0.84
	1-3	-0.15	0.88	25	[-0.32, 0.28]	0.03	0.03	1.49 ± 0.87	1.51 ± 0.82
	1-4	-0.33	0.74	25	[-0.26, 0.19]	0.06	0.06	1.56 ± 0.78	1.59 ± 0.74
	1-5	-1.70	0.10	25	[-0.30, 0.03]	0.33	0.32	1.50 ± 0.72	1.64 ± 0.72
	1-6	-1.75	0.09	25	[-0.29, 0.02]	0.34	0.33	1.36 ± 0.57	1.50 ± 0.59
	1-7	-1.29	0.21	25	[-0.23, 0.05]	0.25	0.25	1.30 ± 0.46	1.39 ± 0.45
	1-8	-0.90	0.38	25	[-0.20, 0.08]	0.18	0.17	1.26 ± 0.40	1.32 ± 0.35
	1-9	-0.76	0.45	25	[-0.17, 0.08]	0.15	0.15	1.20 ± 0.36	1.25 ± 0.28
	1-10	-1.14	0.26	25	[-0.19, 0.05]	0.22	0.22	1.13 ± 0.33	1.20 ± 0.22
	1-11	-1.00	0.33	25	[-0.16, 0.06]	0.20	0.19	1.08 ± 0.29	1.14 ± 0.18
	1-12	-1.17	0.25	25	[-0.16, 0.04]	0.23	0.22	1.04 ± 0.26	1.10 ± 0.13
	1-13	-1.11	0.28	25	[-0.15, 0.04]	0.22	0.21	1.01 ± 0.25	1.06 ± 0.09
	1-14	-0.93	0.36	25	[-0.13, 0.05]	0.18	0.18	0.98 ± 0.23	1.03 ± 0.05
	1-15	-1.07	0.29	25	[-0.13, 0.04]	0.21	0.20	0.95 ± 0.22	1.00 ± 0.00
during trace interval	1-1	1.13	0.27	25	[-0.64, 2.19]	0.22	0.21	2.23 ± 2.91	1.45 ± 1.39
	1-2	1.00	0.33	25	[-0.59, 1.68]	0.20	0.19	2.30 ± 2.59	1.75 ± 0.93
	1-3	1.22	0.23	25	[-0.33, 1.27]	0.24	0.23	2.36 ± 2.02	1.88 ± 0.84
	1-4	1.38	0.18	25	[-0.21, 1.04]	0.27	0.26	2.18 ± 1.63	1.76 ± 0.73
	1-5	1.27	0.21	25	[-0.20, 0.87]	0.25	0.24	2.06 ± 1.51	1.73 ± 0.69
	1-6	1.12	0.28	25	[-0.22, 0.73]	0.22	0.21	1.84 ± 1.29	1.59 ± 0.56
	1-7	1.22	0.23	25	[-0.17, 0.65]	0.24	0.23	1.72 ± 1.11	1.48 ± 0.44
	1-8	1.14	0.27	25	[-0.16, 0.54]	0.22	0.22	1.58 ± 0.95	1.39 ± 0.33
	1-9	1.18	0.25	25	[-0.14, 0.50]	0.23	0.22	1.47 ± 0.84	1.29 ± 0.26
	1-10	1.12	0.27	25	[-0.14, 0.46]	0.22	0.21	1.39 ± 0.76	1.23 ± 0.20
	1-11	1.12	0.27	25	[-0.12, 0.40]	0.22	0.21	1.32 ± 0.68	1.18 ± 0.16
	1-12	1.03	0.31	25	[-0.12, 0.35]	0.20	0.20	1.25 ± 0.61	1.14 ± 0.10
	1-13	1.11	0.28	25	[-0.10, 0.33]	0.22	0.21	1.20 ± 0.55	1.08 ± 0.07
	1-14	1.10	0.28	25	[-0.09, 0.31]	0.22	0.21	1.14 ± 0.51	1.04 ± 0.04
	1-15	1.05	0.30	25	[-0.09, 0.29]	0.21	0.20	1.10 ± 0.47	1.00 ± 0.00
to timepooint of US	1-1	-1.12	0.27	25	[-1.60, 0.47]	0.22	0.21	0.76 ± 1.33	1.33 ± 2.35
	1-2	-0.62	0.54	25	[-0.79, 0.42]	0.12	0.12	1.04 ± 1.33	1.23 ± 1.46
	1-3	-0.60	0.55	25	[-0.65, 0.36]	0.12	0.11	1.03 ± 1.17	1.18 ± 1.26
	1-4	-1.09	0.29	25	[-0.50, 0.15]	0.21	0.21	1.12 ± 1.12	1.29 ± 1.18
	1-5	-1.18	0.25	25	[-0.57, 0.15]	0.23	0.23	1.13 ± 1.04	1.34 ± 1.10
	1-6	-1.49	0.15	25	[-0.52, 0.08]	0.29	0.28	1.00 ± 0.86	1.22 ± 0.91
	1-7	-1.65	0.11	25	[-0.44, 0.05]	0.32	0.31	0.93 ± 0.75	1.12 ± 0.76
	1-8	-1.94	0.06	25	[-0.42, 0.01]	0.38	0.37	0.91 ± 0.61	1.11 ± 0.61
	1-9	-1.42	0.17	25	[-0.37, 0.07]	0.28	0.27	0.92 ± 0.57	1.07 ± 0.49
	1-10	-1.05	0.30	25	[-0.33, 0.11]	0.21	0.20	0.90 ± 0.52	1.01 ± 0.43
	1-11	-2.47	0.021*	25	[-0.39, -0.03]	0.48	0.47	0.87 ± 0.49	1.08 ± 0.30
	1-12	-2.48	0.020*	25	[-0.35, -0.03]	0.49	0.47	0.86 ± 0.44	1.05 ± 0.26
	1-13	-2.45	0.022*	25	[-0.37, -0.03]	0.48	0.47	0.88 ± 0.41	1.08 ± 0.09
	1-14	-1.40	0.17	25	[-0.29, 0.06]	0.27	0.27	0.93 ± 0.43	1.05 ± 0.03
	1-15	-1.22	0.24	25	[-0.26, 0.07]	0.24	0.23	0.90 ± 0.40	1.00 ± 0.00

Table S6

Experiment 2: recall SCR DCM modelled paired t-test, not corrected for multiple comparisons

	trials	t-statistic	p	df	95% CI	cohen's d	hedge's g	Mean (± SD) CS+	Mean (± SD) CS-
to CS presentation	1-1	-0.13	0.90	26	[-0.90, 0.79]	0.02	0.02	1.00 ± 1.40	1.05 ± 0.97
	1-2	0.66	0.52	26	[-0.32, 0.62]	0.13	0.12	1.40 ± 0.92	1.25 ± 0.66
	1-3	0.26	0.80	26	[-0.26, 0.33]	0.05	0.05	1.35 ± 0.72	1.31 ± 0.58
	1-4	0.08	0.93	26	[-0.20, 0.22]	0.02	0.02	1.33 ± 0.66	1.33 ± 0.54
	1-5	-0.22	0.83	26	[-0.20, 0.16]	0.04	0.04	1.31 ± 0.60	1.32 ± 0.51
	1-6	-0.25	0.80	26	[-0.18, 0.14]	0.05	0.05	1.18 ± 0.51	1.20 ± 0.42
	1-7	-0.27	0.79	26	[-0.17, 0.13]	0.05	0.05	1.15 ± 0.44	1.17 ± 0.33
	1-8	-0.06	0.95	26	[-0.14, 0.13]	0.01	0.01	1.14 ± 0.37	1.14 ± 0.27
	1-9	-0.32	0.75	26	[-0.14, 0.10]	0.06	0.06	1.09 ± 0.32	1.11 ± 0.23
	1-10	-0.39	0.70	26	[-0.14, 0.09]	0.08	0.07	1.06 ± 0.29	1.08 ± 0.20
	1-11	-0.92	0.36	26	[-0.16, 0.06]	0.18	0.17	0.99 ± 0.28	1.04 ± 0.14
	1-12	-0.88	0.39	26	[-0.15, 0.06]	0.17	0.16	0.99 ± 0.26	1.04 ± 0.10
	1-13	-1.29	0.21	26	[-0.16, 0.04]	0.25	0.24	0.98 ± 0.24	1.04 ± 0.06
	1-14	-0.94	0.35	26	[-0.14, 0.05]	0.18	0.18	0.98 ± 0.24	1.02 ± 0.04
	1-15	-0.54	0.60	26	[-0.12, 0.07]	0.10	0.10	0.97 ± 0.24	1.00 ± 0.00
during trace interval	1-1	-0.55	0.59	26	[-0.65, 0.38]	0.11	0.10	1.20 ± 1.07	1.34 ± 0.73
	1-2	-0.58	0.57	26	[-0.41, 0.23]	0.11	0.11	1.36 ± 0.81	1.45 ± 0.60
	1-3	-0.59	0.56	26	[-0.30, 0.17]	0.11	0.11	1.40 ± 0.70	1.46 ± 0.53
	1-4	-0.66	0.52	26	[-0.24, 0.13]	0.13	0.12	1.36 ± 0.59	1.42 ± 0.45
	1-5	-0.07	0.94	26	[-0.19, 0.18]	0.01	0.01	1.37 ± 0.61	1.38 ± 0.42
	1-6	-0.02	0.98	26	[-0.16, 0.16]	0.00	0.00	1.31 ± 0.54	1.31 ± 0.31
	1-7	0.53	0.60	26	[-0.10, 0.16]	0.10	0.10	1.28 ± 0.45	1.25 ± 0.27
	1-8	0.48	0.63	26	[-0.09, 0.14]	0.09	0.09	1.24 ± 0.38	1.21 ± 0.22
	1-9	-0.12	0.91	26	[-0.13, 0.11]	0.02	0.02	1.20 ± 0.35	1.21 ± 0.20
	1-10	0.22	0.82	26	[-0.10, 0.13]	0.04	0.04	1.18 ± 0.32	1.17 ± 0.18
	1-11	0.60	0.56	26	[-0.07, 0.13]	0.12	0.11	1.17 ± 0.29	1.14 ± 0.13
	1-12	0.93	0.36	26	[-0.06, 0.15]	0.18	0.17	1.14 ± 0.28	1.10 ± 0.10
	1-13	1.32	0.20	26	[-0.04, 0.17]	0.25	0.25	1.12 ± 0.27	1.06 ± 0.07
	1-14	1.46	0.16	26	[-0.03, 0.17]	0.28	0.27	1.09 ± 0.25	1.02 ± 0.04
	1-15	1.42	0.17	26	[-0.03, 0.16]	0.27	0.27	1.07 ± 0.25	1.00 ± 0.00
to timepooint of US	1-1	1.57	0.13	26	[-0.11, 0.85]	0.30	0.29	0.82 ± 1.28	0.45 ± 1.06
	1-2	1.26	0.22	26	[-0.17, 0.71]	0.24	0.24	0.72 ± 1.16	0.45 ± 0.78
	1-3	1.25	0.22	26	[-0.20, 0.80]	0.24	0.23	0.91 ± 1.26	0.60 ± 0.86
	1-4	1.55	0.13	26	[-0.14, 0.97]	0.30	0.29	1.01 ± 1.37	0.59 ± 0.77
	1-5	1.78	0.087	26	[-0.06, 0.90]	0.34	0.33	1.03 ± 1.18	0.61 ± 0.73
	1-6	1.07	0.30	26	[-0.18, 0.58]	0.20	0.20	0.89 ± 0.99	0.69 ± 0.60
	1-7	0.95	0.35	26	[-0.22, 0.60]	0.18	0.18	0.95 ± 1.03	0.76 ± 0.50
	1-8	0.76	0.45	26	[-0.23, 0.50]	0.15	0.14	0.93 ± 0.95	0.80 ± 0.47
	1-9	1.36	0.18	26	[-0.18, 0.90]	0.26	0.25	1.17 ± 1.31	0.81 ± 0.41
	1-10	1.91	0.067	26	[-0.04, 1.00]	0.37	0.36	1.28 ± 1.16	0.80 ± 0.37
	1-11	1.58	0.13	26	[-0.13, 0.99]	0.30	0.30	1.27 ± 1.28	0.84 ± 0.35
	1-12	1.21	0.24	26	[-0.20, 0.78]	0.23	0.23	1.26 ± 1.17	0.97 ± 0.25
	1-13	1.64	0.11	26	[-0.14, 1.28]	0.32	0.31	1.57 ± 1.78	1.01 ± 0.12
	1-14	1.57	0.13	26	[-0.16, 1.21]	0.30	0.29	1.54 ± 1.72	1.02 ± 0.08
	1-15	1.64	0.11	26	[-0.13, 1.14]	0.32	0.31	1.50 ± 1.60	1.00 ± 0.00

Table S7

Experiment 1: recall PSR standard RF, paired t-test, not corrected for multiple comparisons

trials	t-statistic	p	df	95% CI	cohen's d	hedge's g	Mean (± SD) CS+	Mean (± SD) CS-
1-1	1.31	0.20	27	[-0.02, 0.11]	0.25	0.24	1.20 ± 0.16	1.15 ± 0.10
1-2	1.03	0.31	27	[-0.02, 0.07]	0.19	0.19	1.12 ± 0.10	1.10 ± 0.07
1-3	0.68	0.50	27	[-0.02, 0.04]	0.13	0.13	1.08 ± 0.08	1.07 ± 0.04
1-4	0.28	0.78	27	[-0.03, 0.04]	0.05	0.05	1.04 ± 0.08	1.04 ± 0.04
1-5	0.11	0.92	27	[-0.03, 0.03]	0.02	0.02	1.03 ± 0.07	1.03 ± 0.03
1-6	0.13	0.90	27	[-0.02, 0.03]	0.02	0.02	1.03 ± 0.06	1.02 ± 0.03
1-7	-0.60	0.55	27	[-0.03, 0.02]	0.11	0.11	1.01 ± 0.05	1.02 ± 0.03
1-8	-0.30	0.76	27	[-0.02, 0.02]	0.06	0.06	1.01 ± 0.05	1.02 ± 0.02
1-9	-0.16	0.87	27	[-0.02, 0.02]	0.03	0.03	1.01 ± 0.05	1.01 ± 0.02
1-10	-0.17	0.87	27	[-0.02, 0.02]	0.03	0.03	1.01 ± 0.04	1.01 ± 0.02
1-11	-0.27	0.79	27	[-0.02, 0.01]	0.05	0.05	1.01 ± 0.04	1.01 ± 0.02
1-12	0.08	0.94	27	[-0.01, 0.01]	0.02	0.02	1.01 ± 0.04	1.01 ± 0.02
1-13	0.17	0.87	27	[-0.01, 0.01]	0.03	0.03	1.01 ± 0.03	1.00 ± 0.01
1-14	0.71	0.48	27	[-0.01, 0.02]	0.13	0.13	1.00 ± 0.03	1.00 ± 0.01
1-15	0.90	0.37	27	[-0.01, 0.02]	0.17	0.17	1.01 ± 0.03	1.00 ± 0.00

Table S8

Experiment 2: recall PSR standard RF, paired t-test, not corrected for multiple comparisons

trials	t-statistic	p	df	95% CI	cohen's d	hedge's g	Mean (± SD) CS+	Mean (± SD) CS-
1-1	-0.35	0.73	27	[-0.07, 0.05]	0.07	0.06	1.15 ± 0.18	1.16 ± 0.12
1-2	-0.39	0.70	27	[-0.06, 0.04]	0.07	0.07	1.10 ± 0.13	1.10 ± 0.08
1-3	-0.07	0.94	27	[-0.04, 0.04]	0.01	0.01	1.06 ± 0.09	1.06 ± 0.06
1-4	-0.11	0.92	27	[-0.03, 0.03]	0.02	0.02	1.04 ± 0.07	1.04 ± 0.05
1-5	-0.65	0.52	27	[-0.03, 0.02]	0.12	0.12	1.02 ± 0.06	1.02 ± 0.04
1-6	-0.33	0.75	27	[-0.02, 0.02]	0.06	0.06	1.02 ± 0.05	1.02 ± 0.04
1-7	0.09	0.93	27	[-0.02, 0.02]	0.02	0.02	1.01 ± 0.06	1.01 ± 0.04
1-8	-0.30	0.77	27	[-0.02, 0.02]	0.06	0.05	1.01 ± 0.05	1.01 ± 0.03
1-9	-0.93	0.36	27	[-0.02, 0.01]	0.18	0.17	1.00 ± 0.04	1.01 ± 0.02
1-10	-0.87	0.39	27	[-0.02, 0.01]	0.16	0.16	1.00 ± 0.04	1.00 ± 0.02
1-11	-0.26	0.80	27	[-0.02, 0.01]	0.05	0.05	1.00 ± 0.04	1.00 ± 0.02
1-12	-0.71	0.48	27	[-0.02, 0.01]	0.13	0.13	1.00 ± 0.04	1.00 ± 0.01
1-13	-0.90	0.37	27	[-0.02, 0.01]	0.17	0.17	1.00 ± 0.03	1.00 ± 0.01
1-14	-0.98	0.33	27	[-0.02, 0.01]	0.19	0.18	1.00 ± 0.03	1.00 ± 0.01
1-15	-0.63	0.53	27	[-0.02, 0.01]	0.12	0.12	1.00 ± 0.03	1.00 ± 0.00

Table S9

Experiment 1: recall PSR fitted RF, paired t-test, not corrected for multiple comparisons

							Mean (± SD)	
**trials**	**t-statistic**	**p**	**df**	**95% CI**	**cohen's d**	**hedge's g**	**CS+**	**CS-**
1-1	2.03	0.052	27	[0.00, 0.33]	0.38	0.37	1.53 ± 0.39	1.37 ± 0.31
1-2	1.20	0.24	27	[-0.05, 0.19]	0.23	0.22	1.34 ± 0.25	1.27 ± 0.25
1-3	1.46	0.16	27	[-0.03, 0.20]	0.28	0.27	1.22 ± 0.23	1.14 ± 0.19
1-4	1.22	0.23	27	[-0.04, 0.16]	0.23	0.22	1.13 ± 0.19	1.07 ± 0.13
1-5	1.33	0.19	27	[-0.04, 0.17]	0.25	0.24	1.09 ± 0.19	1.02 ± 0.13
1-6	1.11	0.28	27	[-0.04, 0.13]	0.21	0.20	1.06 ± 0.16	1.01 ± 0.11
1-7	0.65	0.52	27	[-0.05, 0.10]	0.12	0.12	1.03 ± 0.16	1.01 ± 0.08
1-8	0.84	0.41	27	[-0.04, 0.10]	0.16	0.15	1.03 ± 0.15	1.00 ± 0.06
1-9	0.84	0.41	27	[-0.03, 0.08]	0.16	0.16	1.02 ± 0.13	1.00 ± 0.05
1-10	0.70	0.49	27	[-0.03, 0.07]	0.13	0.13	1.02 ± 0.12	1.00 ± 0.05
1-11	0.52	0.61	27	[-0.04, 0.06]	0.10	0.10	1.02 ± 0.12	1.01 ± 0.03
1-12	0.10	0.92	27	[-0.04, 0.04]	0.02	0.02	1.01 ± 0.11	1.01 ± 0.03
1-13	0.62	0.54	27	[-0.03, 0.05]	0.12	0.11	1.02 ± 0.11	1.01 ± 0.03
1-14	0.88	0.39	27	[-0.02, 0.06]	0.17	0.16	1.02 ± 0.10	1.00 ± 0.02
1-15	1.37	0.18	27	[-0.01, 0.07]	0.26	0.25	1.03 ± 0.11	1.00 ± 0.00

Table S10

Experiment 2: recall PSR fitted RF, paired t-test, not corrected for multiple comparisons

							Mean (± SD)	
**trials**	**t-statistic**	**p**	**df**	**95% CI**	**cohen's d**	**hedge's g**	**CS+**	**CS-**
1-1	0.33	0.75	27	[-0.14, 0.19]	0.06	0.06	1.48 ± 0.58	1.45 ± 0.43
1-2	0.65	0.52	27	[-0.07, 0.14]	0.12	0.12	1.35 ± 0.42	1.31 ± 0.28
1-3	0.86	0.40	27	[-0.04, 0.11]	0.16	0.16	1.21 ± 0.27	1.18 ± 0.17
1-4	1.18	0.25	27	[-0.03, 0.10]	0.22	0.22	1.13 ± 0.18	1.10 ± 1.15
1-5	0.59	0.56	27	[-0.04, 0.06]	0.11	0.11	1.05 ± 0.14	1.04 ± 0.11
1-6	-0.12	0.90	27	[-0.06, 0.06]	0.02	0.02	1.05 ± 0.14	1.06 ± 0.12
1-7	0.40	0.69	27	[-0.06, 0.08]	0.08	0.07	1.05 ± 0.15	1.04 ± 0.12
1-8	0.28	0.78	27	[-0.05, 0.07]	0.05	0.05	1.04 ± 0.15	1.04 ± 0.09
1-9	-0.09	0.93	27	[-0.05, 0.05]	0.02	0.02	1.03 ± 0.13	1.03 ± 0.09
1-10	0.18	0.86	27	[-0.04, 0.05]	0.03	0.03	1.01 ± 0.13	1.01 ± 0.08
1-11	-0.05	0.96	27	[-0.05, 0.05]	0.01	0.01	1.02 ± 0.14	1.02 ± 0.06
1-12	0.43	0.67	27	[-0.04, 0.06]	0.08	0.08	1.02 ± 0.14	1.01 ± 0.05
1-13	0.51	0.62	27	[-0.04, 0.07]	0.10	0.09	1.01 ± 0.15	1.00 ± 0.04
1-14	0.01	0.99	27	[-0.06, 0.06]	0.00	0.00	1.00 ± 0.14	1.00 ± 0.02
1-15	0.07	0.94	27	[-0.05, 0.06]	0.01	0.01	1.00 ± 0.14	1.00 ± 0.00

Table S11

Experiment 1: recall SF, paired t-test, not corrected for multiple comparisons

							Mean (± SD)	
**trials**	**t-statistic**	**p**	**df**	**95% CI**	**cohen's d**	**hedge's g**	**CS+**	**CS-**
1-1	0.49	0.63	27	[-0.93, 1.52]	0.09	0.09	3.23 ± 3.93	2.94 ± 3.14
1-2	1.06	0.30	27	[-0.35, 1.10]	0.20	0.19	2.45 ± 2.33	2.08 ± 1.75
1-3	0.93	0.36	27	[-0.28, 0.74]	0.18	0.17	2.01 ± 1.69	1.78 ± 1.19
1-4	0.63	0.54	27	[-0.26, 0.50]	0.12	0.12	1.74 ± 1.23	1.63 ± 0.88
1-5	0.40	0.69	27	[-0.25, 0.37]	0.08	0.07	1.54 ± 0.97	1.48 ± 0.72
1-6	0.42	0.68	27	[-0.24, 0.37]	0.08	0.08	1.45 ± 0.86	1.39 ± 0.57
1-7	0.50	0.62	27	[-0.17, 0.28]	0.09	0.09	1.38 ± 0.73	1.33 ± 0.47
1-8	0.72	0.48	27	[-0.13, 0.27]	0.14	0.13	1.32 ± 0.64	1.25 ± 0.36
1-9	0.62	0.54	27	[-0.12, 0.23]	0.12	0.11	1.25 ± 0.56	1.20 ± 0.29
1-10	0.35	0.73	27	[-0.13, 0.19]	0.07	0.06	1.19 ± 0.49	1.17 ± 0.25
1-11	0.91	0.37	27	[-0.09, 0.23]	0.17	0.17	1.19 ± 0.47	1.12 ± 0.21
1-12	1.09	0.28	27	[-0.07, 0.23]	0.21	0.20	1.16 ± 0.43	1.09 ± 0.17
1-13	0.74	0.46	27	[-0.09, 0.19]	0.14	0.14	1.12 ± 0.40	1.07 ± 0.10
1-14	0.84	0.41	27	[-0.08, 0.20]	0.16	0.16	1.08 ± 0.39	1.03 ± 0.07
1-15	0.82	0.42	27	[-0.09, 0.20]	0.16	0.15	1.06 ± 0.37	1.00 ± 0.00

Table S12

Experiment 2: recall SF, paired t-test, not corrected for multiple comparisons

							Mean (± SD)	
**trials**	**t-statistic**	**p**	**df**	**95% CI**	**cohen's d**	**hedge's g**	**CS+**	**CS-**
1-1	0.67	0.51	27	[-0.61, 1.20]	0.13	0.12	2.43 ± 2.11	2.14 ± 1.52
1-2	0.88	0.39	27	[-0.36, 0.89]	0.17	0.16	2.04 ± 1.56	1.78 ± 1.18
1-3	0.93	0.36	27	[-0.24, 0.63]	0.18	0.17	1.65 ± 1.09	1.46 ± 0.85
1-4	1.08	0.29	27	[-0.16, 0.51]	0.20	0.20	1.49 ± 0.81	1.32 ± 0.69
1-5	0.57	0.57	27	[-0.22, 0.39]	0.11	0.11	1.33 ± 0.70	1.25 ± 0.54
1-6	1.27	0.21	27	[-0.13, 0.56]	0.24	0.23	1.41 ± 0.82	1.20 ± 0.45
1-7	1.29	0.21	27	[-0.12, 0.51]	0.24	0.24	1.33 ± 0.73	1.13 ± 0.35
1-8	1.38	0.18	27	[-0.09, 0.46]	0.26	0.25	1.28 ± 0.66	1.09 ± 0.31
1-9	1.09	0.29	27	[-0.11, 0.37]	0.21	0.20	1.21 ± 0.58	1.08 ± 0.24
1-10	1.10	0.28	27	[-0.10, 0.34]	0.21	0.20	1.16 ± 0.55	1.04 ± 0.21
1-11	1.65	0.11	27	[-0.04, 0.37]	0.31	0.30	1.21 ± 0.51	1.04 ± 0.16
1-12	1.43	0.16	27	[-0.06, 0.33]	0.27	0.26	1.17 ± 0.48	1.03 ± 0.14
1-13	1.17	0.25	27	[-0.08, 0.28]	0.22	0.21	1.14 ± 0.44	1.03 ± 0.09
1-14	1.30	0.20	27	[-0.06, 0.27]	0.25	0.24	1.13 ± 0.42	1.02 ± 0.04
1-15	1.40	0.17	27	[-0.05, 0.26]	0.26	0.26	1.11 ± 0.41	1.00 ± 0.00

Table S13

Acquisition paired t-test CS+/CS-, not corrected for multiple comparisons

Measure	EXP	Specification	trials	t-statistic	p	df	95% CI	cohen's d	Mean (± SD) CS+	Mean (± SD) CS-
SCR DCM	1	to CS presentation	1-20	3.14	0.004*	25	[0.07, 0.33]	0.22	1.20 ± 0.32	1.00 ± 0.00
		during trace interval	1-20	3.87	0.001*	25	[0.29, 0.97]	0.43	1.63 ± 0.83	1.00 ± 0.00
		to US presentation	1-20	5.12	<.001*	25	[0.64, 1.50]	0.52	2.07 ± 1.07	1.00 ± 0.00
	2	to CS presentation	1-20	3.12	0.004*	26	[0.07, 0.35]	0.20	1.21 ± 0.35	1.00 ± 0.00
		during trace interval	1-20	3.62	0.001*	26	[0.23, 0.83]	0.32	1.53 ± 0.76	1.00 ± 0.00
		to US presentation	1-20	4.24	<.001*	26	[0.53, 1.53]	0.38	2.03 ± 1.26	1.00 ± 0.00
SF	1		1-20	2.60	0.015*	27	[0.05, 0.43]	0.49	1.24 ± 0.49	1.00 ± 0.00
	2		1-20	2.55	0.017*	27	[0.06, 0.57]	0.48	1.31 ± 0.65	1.00 ± 0.00
PSR	1	standard	1-20	3.10	0.004*	27	[0.01, 0.03]	0.59	1.02 ± 0.03	1.00 ± 0.00
		fitted	1-20	8.20	< .001*	27	[0.19, 0.32]	1.55	1.26 ± 0.17	1.00 ± 0.00
	2	standard	1-20	1.34	0.19	27	[-0.01, 0.03]	0.25	1.01 ± 0.04	1.00 ± 0.00
		fitted	1-20	5.94	< .001*	27	[0.19, 0.39]	1.12	1.29 ± 0.26	1.00 ± 0.00

Table S14

Paired t-test CS+/CS-, not corrected for multiple comparisons

Measure	Exp	Session	trials	t-statistic	p	df	95% CI	cohen's d	Mean (± SD) CS+	Mean (± SD) CS-
HP standard	1	acquisition	1-20	1.97	0.060	27	[-1.62, 75.30]	0.37	48.89 ± 98.41	12.04 ± 22.78
		recall	1-15	-0.21	0.83	27	[-10.48, 8.51]	-0.04	3.02 ± 28.63	4.01 ± 30.64
	2	acquisition	1-20	-0.49	0.63	27	[-33.27, 20.42]	-0.09	7.96 ± 66.67	14.38 ± 17.28
		recall	1-15	-0.43	0.67	27	[-7.66, 5.01]	-0.08	0.47 ± 23.70	1.79 ± 17.59
HP fitted	1	acquisition	1-20	-0.79	0.43	27	[-17.46, 7.72]	-0.15	1.01 ± 30.95	5.88 ± 19.37
		recall	1-15	2.16	0.040*	27	[0.43, 16.37]	0.41	12.61 ± 23.30	4.21 ± 19.13
	2	acquisition	1-20	1.73	0.10	27	[-1.81, 20.99]	0.33	16.60 ± 28.29	7.01 ± 18.76
		recall	1-15	0.12	0.91	27	[-8.36, 9.35]	0.02	13.14 ± 20.08	12.65 ± 20.19
RA	1	acquisition	1-20	1.90	0.068	27	[-0.01, 0.33]	-0.36	-0.12 ± 0.52	0.04 ± 0.45
		recall	1-15	3.55	0.001*	27	[0.08, 0.30]	-0.67	-0.33 ± 0.54	-0.14 ± 0.49
	2	acquisition	1-20	0.77	0.45	27	[-0.08, 0.17]	-0.15	-0.10 ± 0.24	-0.06 ± 0.22
		recall	1-15	0.30	0.77	27	[-0.10, 0.13]	-0.06	-0.12 ± 0.30	-0.10 ± 0.22

Table S15

Experiment 1: SCR DCM estimated response peak during trace interval in s (from start of trial)

		Mean Peak Latency (± SD)	
**Session**	**trial**	**CS+**	**CS-**
Acquisition	1	12.29 ± 2.31	11.38 ± 1.50
	2	12.60 ± 2.74	11.11 ± 1.51
	3	12.03 ± 2.69	10.85 ± 1.41
	4	11.33 ± 2.17	10.80 ± 1.19
	5	11.86 ± 2.52	10.74 ± 1.14
	6	11.31 ± 2.21	10.63 ± 1.01
	7	11.78 ± 2.46	11.13 ± 1.53
	8	12.26 ± 2.57	10.81 ± 1.56
	9	11.67 ± 2.39	11.01 ± 1.43
	10	12.04 ± 2.41	11.43 ± 1.82
	11	11.88 ±2.20	10.56 ± 1.00
	12	11.44 ± 2.08	10.86 ± 1.13
	13	11.13 ± 1.89	11.32 ± 1.64
	14	11.44 ± 2.17	10.98 ± 1.49
	15	10.84 ± 1.46	10.92 ± 1.18
	16	11.69 ± 2.12	11.21 ± 1.52
	17	11.60 ± 2.09	11.12 ± 1.50
	18	11.57 ± 2.07	10.77 ± 1.45
	19	11.00 ± 1.72	11.44 ± 1.85
	20	11.13 ± 1.98	11.02 ± 1.28
Recall	1	12.47 ± 1.83	12.93 ± 1.61
	2	11.99 ± 1.92	12.09 ± 1.68
	3	12.17 ± 1.99	12.42 ± 1.85
	4	12.26 ± 1.86	11.59 ± 1.91
	5	12.14 ± 1.91	11.81 ± 1.88
	6	12.32 ± 1.60	12.07 ± 1.88
	7	12.70 ± 1.90	12.38 ± 1.76
	8	12.47 ± 2.01	12.32 ± 2.01
	9	11.93 ± 1.87	12.01 ± 1.92
	10	11.71 ± 1.90	12.11 ± 1.84
	11	12.39 ± 2.02	11.88 ± 1.83
	12	11.98 ± 1.79	12.26 ± 2.01
	13	11.69 ± 1.85	11.22 ± 1.70
	14	11.65 ± 1.95	11.43 ± 1.83
	15	11.38 ± 1.65	12.54 ± 2.13

Table S16

Experiment 2: SCR DCM estimated response peak during trace interval in s (from start of trial)

		Mean Peak Latency (± SD)	
**Session**	**trial**	**CS+**	**CS-**
Acquisition	1	12.32 ± 2.57	11.50 ± 1.87
	2	12.47 ± 2.52	11.37 ± 1.87
	3	11.23 ± 1.90	10.75 ± 1.20
	4	11.80 ± 2.41	10.87 ± 1.25
	5	11.55 ± 2.30	10.85 ± 1.37
	6	11.69 ± 2.20	10.76 ± 1.12
	7	10.78 ± 1.76	11.03 ± 1.43
	8	11.22 ± 1.98	10.90 ± 1.46
	9	10.95 ± 1.87	10.48 ± 0.88
	10	11.31 ± 1.88	10.69 ± 1.22
	11	11.45 ± 2.02	11.25 ± 1.42
	12	11.26 ± 1.89	11.35 ± 1.44
	13	10.85 ± 1.55	10.45 ± 0.78
	14	10.94 ± 1.43	11.11 ± 1.59
	15	11.34 ± 2.01	11.38 ± 1.63
	16	12.38 ± 2.64	10.86 ± 1.25
	17	10.69 ± 1.33	10.71 ± 1.17
	18	11.44 ± 2.17	11.03 ± 1.67
	19	10.98 ± 1.84	11.22 ± 1.39
	20	12.53 ± 2.64	10.78 ± 1.24
Recall	1	12.29 ± 1.67	12.68 ± 1.52
	2	12.66 ± 1.49	12.68 ± 1.88
	3	12.84 ± 1.54	13.08 ± 1.51
	4	12.32 ± 1.75	12.64 ± 1.74
	5	12.22 ± 1.81	12.57 ± 1.78
	6	12.76 ± 1.62	12.36 ± 1.76
	7	12.18 ± 1.86	12.34 ± 1.83
	8	12.38 ± 1.73	11.97 ± 2.01
	9	12.00 ± 1.79	12.07 ± 1.93
	10	11.95 ± 1.89	12.60 ± 1.71
	11	12.36 ± 1.74	12.58 ± 1.81
	12	12.86 ± 1.89	12.77 ± 1.81
	13	12.39 ± 1.78	11.88 ± 1.74
	14	12.26 ± 1.87	11.59 ± 1.70
	15	11.63 ± 1.80	12.55 ± 2.02

Table S17

Experiment 1: SCR LME in recall

lmer(data ~ (1|subject)+trial *condition)

	to CS					Trace Interval				
	F-value	df	p-value	90% CI	η^2	F-value	df	p-value	90% CI	η^2
**Trial number**	166.00	1, 809	<.001*	[0.13, 0.20]	0.16	205.45	1, 809	<.001*	[0.16, 0.24]	0.21
**Condition (CS+/CS-)**	0.24	1, 809	0.624	[0.00, 0.01]	<.01	3.45	1, 809	0.064	[0.00, 0.01]	0.02
**Condition x trial**	0.11	1, 809	0.741	[0.00, 0.00]	<.01	9.79	1, 809	0.002*	[0.00, 0.03]	0.01
**AIC**	2102.56					2729.88				

lmer(data ~ (1+trial|subject)+trial *condition)

	to CS					Trace Interval				
	F-value	df	p-value	90% CI	η^2	F-value	df	p-value	90% CI	η^2
**Trial number**	23.22	1, 809	<.001*	[0.01, 0.05]	0.460	35.28	1, 809	<.001*	[0.02, 0.07]	0.57
**Condition (CS+/CS-)**	0.07	1, 809	0.795	[0.00, 0.00]	<.01	5.53	1, 809	0.019*	[0.00, 0.02]	0.02
**Condition x trial**	0.12	1, 809	0.734	[0.00 0.00]	<.01	10.94	1, 809	<.001*	[0.00, 0.03]	0.01
**AIC**	1973.93					2625.52				

Table S18

Experiment 2: SCR LME in recall

lmer(data ~ (1+trial|subject)+trial *condition)

	to CS					Trace Interval				
	F-value	df	p-value	90% CI	η^2	F-value	df	p-value	90% CI	η^2
**Trial number**	8.54	1, 780	0.004*	[0.00, 0.03]	0.25	21.01	1, 780	<.001*	[0.01, 0.05]	0.45
**Condition (CS+/CS-)**	0.26	1, 780	0.61	[0.00, 0.01]	<.01	1.90	1, 780	0.17	[0.00, 0.01]	<.01
**Condition x trial**	0.04	1, 780	0.84	[0.00, 0.00]	<.01	1.55	1, 780	0.21	[0.00, 0.01]	<.01

Table S19

Experiment 1 and 2 combined: SCR LME in recall

lmer(data ~ (1+trial|subject)+trial *condition)

	Trace Interval				
	F-value	df	p-value	90% CI	η^2
**Trial number**	42.84	1, 1563	<.001*	[0.01, 0.04]	0.45
**Condition (CS+/CS-)**	3.80	1, 1563	0.051	[0.00, 0.01]	<.01
**Condition x trial**	3.10	1, 1563	0.078	[0.00, 0.01]	<.01

Table S20

Experiment 1: SEBR GLM LME in recall

lmer(data ~ (1|subject)+ $e^{((-1*fit)*trial)}$ *condition)

	F-value	df	p-value	90% CI	η^2
**Trial number**	115.25	1, 722	<.001*	[0.10, 0.17]	0.13
**Condition (CS+/CS-)**	4.42	1, 722	0.036*	[0.00, 0.02]	<.01
**Condition x trial**	1.80	1, 722	0.180	[0.00, 0.01]	<.01
**AIC**	2359.42				

lmer(data ~ (1+trial|subject)+$e^{((-1*fit)*trial)}$ *condition)

	F-value	df	p-value	90% CI	η^2
**Trial number**	47.45	1, 722	<.001*	[0.03, 0.09]	0.66
**Condition (CS+/CS-)**	4.68	1, 722	0.031*	[0.00, 0.02]	0.01
**Condition x trial**	1.97	1, 722	0.161	[0.00, 0.01]	<.01
**AIC**	2346.66				

Table S21

Experiment 2: SEBR GLM LME in recall

lmer(data ~ (1+trial|subject)+$e^{((-1*fit)*trial)}$ *condition)

	F-value	df	p-value	90% CI	η^2
**Trial number**	28.80	1, 751	<.001*	[0.02, 0.06]	0.42
**Condition (CS+/CS-)**	8.74	1, 751	0.003*	[0.00, 0.03]	<.01
**Condition x trial**	0.08	1, 751	0.772	[0.00, 0.00]	<.01

Table S22

Experiment 1: SEBR peak scoring LME in recall

lmer(data ~ (1|subject)+$e^{((-1*fit)*trial)}$ *condition)

	F-value	df	p-value	90% CI	η^2
**Trial number**	199.33	1, 722	<.001*	[0.17, 0.25]	0.21
**Condition (CS+/CS-)**	4.93	1, 722	0.027*	[0.00, 0.02]	<.01
**Condition x trial**	1.82	1, 722	0.178	[0.00, 0.01]	<.01
**AIC**	1474.02				

lmer(data ~ (1+trial|subject)+$e^{((-1*fit)*trial)}$ *condition)

	F-value	df	p-value	90% CI	η^2
**Trial number**	61.84	1, 722	<.001*	[0.05, 0.11]	0.72
**Condition (CS+/CS-)**	5.48	1, 722	0.020*	[0.00, 0.02]	0.01
**Condition x trial**	1.92	1, 722	0.167	[0.00, 0.01]	<.01
**AIC**	1449.83				

Table S23

Experiment 2: SEBR peak scoring LME in recall

lmer(data ~ (1+trial|subject)+$e^{((-1*fit)*trial)}$ *condition)

	F-value	df	p-value	90% CI	η^2
**Trial number**	80.03	1, 751	<.001*	[0.06, 0.13]	0.76
**Condition (CS+/CS-)**	6.04	1, 751	0.014*	[0.00, 0.02]	0.01
**Condition x trial**	1.08	1, 751	0.300	[0.00, 0.01]	<.01

Table S24

Experiment 1: SEBR peak scoring ANOVA in recall

aov(data ~ trial x condition)

	F-value	df	p-value	90% CI	η^2	cohen's f
**Trial number**	7.43	29, 690	<.001*	[0.16, 0.24]	0.23	0.56
**Condition (CS+/CS-)**	4.77	1, 690	0.029*	[0.00, 0.02]	0.01	0.08
**Condition x trial**	0.80	29, 690	0.759	[0.00, 0.01]	0.02	0.18

Table S25

Experiment 2: SEBR peak scoring ANOVA in recall

aov(data ~ trial x condition)

	F-value	df	p-value	90% CI	η^2	cohen's f
**Trial number**	10.05	29, 720	<.001*	[0.21, 0.30]	0.28	0.64
**Condition (CS+/CS-)**	7.19	1, 720	0.008*	[0.00, 0.02]	0.01	0.10
**Condition x trial**	0.97	29, 720	0.506	[0.00, 0.02]	0.03	0.20
